# Mild-Temperature Supercritical Water Confined in Hydrophobic Metal–Organic Frameworks

**DOI:** 10.1021/jacs.4c01226

**Published:** 2024-05-03

**Authors:** Sebastiano Merchiori, Andrea Le Donne, Josh D. Littlefair, Alexander Rowland Lowe, Jiang-Jing Yu, Xu-Dong Wu, Mian Li, Dan Li, Monika Geppert-Rybczyńska, Lukasz Scheller, Benjamin A. Trump, Andrey A. Yakovenko, Paweł Zajdel, Mirosław Chorążewski, Yaroslav Grosu, Simone Meloni

**Affiliations:** † Department of Chemical, Pharmaceutical and Agricultural Sciences, University of Ferrara, 44121 Ferrara, Italy; ‡ Institute of Chemistry, University of Silesia, Szkolna 9, 40-006 Katowice, Poland; § College of Chemistry and Chemical Engineering, and Chemistry and Chemical Engineering Guangdong Laboratory, Shantou University, Guangdong 515063, China; ∥ College of Chemistry and Materials Science, Jinan University, Guangzhou 510632, China; ⊥ Institute of Physics, University of Silesia, 41-500 Chorzów, Poland; # Centre for Cooperative Research on Alternative Energies (CIC energiGUNE), Basque Research and Technology Alliance (BRTA), 01510 Vitoria-Gasteiz, Spain; ∇ NIST Center for Neutron Research, National Institute of Standards and Technology, Gaithersburg, Maryland 20899, United States; ○ X-ray Science Division, Advanced Photon Source, Argonne National Laboratory, Argonne, Illinois 60439, United States

## Abstract

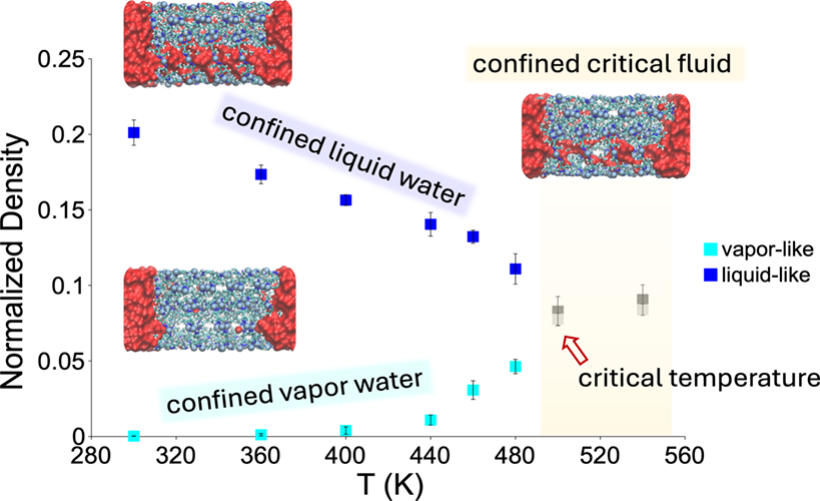

Fluids under extreme confinement show characteristics significantly different from those of their bulk counterpart. This work focuses on water confined within the complex cavities of highly hydrophobic metal–organic frameworks (MOFs) at high pressures. A combination of high-pressure intrusion–extrusion experiments with molecular dynamic simulations and synchrotron data reveals that supercritical transition for MOF-confined water takes place at a much lower temperature than in bulk water, ∼250 K below the reference values. This large shifting of the critical temperature (*T*_c_) is attributed to the very large density of confined water vapor in the peculiar geometry and chemistry of the cavities of Cu_2_tebpz (tebpz = 3,3′,5,5′-tetraethyl-4,4′-bipyrazolate) hydrophobic MOF. This is the first time the shift of *T*_c_ is investigated for water confined within highly hydrophobic nanoporous materials, which explains why such a large reduction of the critical temperature was never reported before, neither experimentally nor computationally.

## 1 Introduction

Force wetting of lyophobic porous solids is nothing but simple. Under ambient conditions, when fully immersed in liquids, their cavities are occupied by gas phases, immiscible liquified gases, or liquid vapor.

Upon application of appropriate perturbations, such as pressure or temperature, the liquid wets the internal cavities of the porous material. This wetting process is commonly denoted as liquid intrusion, while the reverse process is dubbed extrusion. The reversibility of the intrusion process is not guaranteed due to potential free energy barriers that separate the intruded and extruded states, resulting in an intrusion/extrusion hysteresis.^1−4^ The conditions and hysteresis associated with intrusion and extrusion are contingent upon the properties of the porous material and the liquid involved. Understanding the effect of thermodynamic conditions on the intrusion/extrusion process, in the following dubbed liquid porosimetry, and the characteristic of the intruded system at large is not only important per se, but it is also crucial for the technological applications of these heterogeneous lyophobic systems (HLS). Intrusion/extrusion of liquids in porous systems is important for many technological applications, such as the separation of liquids,^5,6^ liquid-phase chromatography,^7,8^ energy damping and storage,^9,10^ porosimetry for the characterization of the porous systems,^11−13^ biological and bioinspired channels,^14−17^ negative compressibility,^18,19^ and liquid piston.^20^ Thus, progress in the understanding of intrusion/extrusion of liquids in porous systems, especially those characterized by cavities of (strictly) nanoscopic size, may unlock novel technological applications. Hydrophobic metal–organic frameworks (MOFs), with internal areas considerably larger than those of hydrophobized porous silica (coated with hydrophobic groups), have attracted the interest of the research community. High porosity, facile fabrication, stability, and large hydrophobicity are the characteristics that pave the way for applications requiring cyclic intrusion/extrusion, including, e.g., HLSs consisting of {porous material + water} for mechanical and thermal energy storage.^13−20,25−35^

In the last 50 years, numerous theoretical and computational studies have been made to describe the behavior of water, and more generally of liquids and fluids, under conditions of high confinement. They investigated the effect of temperature and pressure, as well as the morphological/topological and chemical-physical characteristics, such as hydrophobicity/hydrophilicity of the confining material.^21−40^ More recently, experimental studies have also reported changes in the physicochemical properties of water in nanoscale confinement.^21,22,26,41−43^ However, experimental examples of systems consisting of complex materials are few, especially regarding evidence of high pressure and temperature effects.

In this work, we performed a combined experimental and theoretical analysis of water confined within Cu_2_tebpz (tebpz = 3,3′,5,5′-tetraethyl-4,4′-bipyrazolate) and ZIF-8 (Zn(mIm)_2,_ mIm = 2-methylimidazolate) MOFs as a function of temperature. For Cu_2_(tebpz),^44,45^ which, thanks to its thermal stability, allowed us to explore a broader range, liquid porosimetry revealed a sizable decrease of the intrusion volume with increasing temperature. Namely, the intrusion volume becomes negligible at ∼440 K. A careful analysis of the liquid porosimetry results, supported by atomistic simulations, revealed that this reduction of the intrusion volume is due to a large shift of the (confined) critical temperature (*T*_c_), ∼250 K lower than its bulk value. We postulate that this is induced by a surprisingly high vapor density in the porous system. We attribute this to the complex chemistry and topology of the porosities of Cu_2_(tebpz), which, while overall hydrophobic, contains copper sites favoring high local water density for the fluid in the confined gas phase. This phenomenon, suggested by atomistic simulations, is confirmed by synchrotron experiments. Due to stability issues of ZIF-8, we could not reach high enough temperatures to observe such a phenomenon, but the trend of the intrusion volume with temperature suggests that this second MOF presents a similar phenomenology. To the best of our knowledge, neither direct experimental or theoretical evidence of a reduction of the critical temperature of ∼250 K has been reported nor have experimental results been ascribed to such a large shift of *T*_c_.

The effect of confinement on the critical temperature has been discussed in the literature by several authors.^23,46−49^ Most of these studies investigated ambient condition gases or low-temperature boiling liquids. A characteristic of these fluids is that their critical pressure is lower than the saturation pressure. This prevents condensation in the bulk phase outside the porous system before supercriticality is reached. Finally, confinement media are typically lyophilic porous solids, favoring capillary condensation at low pressure; this prevents the bulk fluid from outside the porous system from reaching saturation conditions. These systems were investigated by observing the dependence of pressure–volume isotherms as a function of temperature in capillary condensation experiments (see a more detailed discussion in Section 2). In particular, confined supercriticality corresponds to the temperature at which one observes a change in the plateau slope of the curves,^48^ as discussed in Section 2. Liquid porosimetry used in the present work is an extension of this well-established approach that enables us to overcome its limitations, i.e. water presents a critical pressure much higher than its saturation value. Moreover, liquid porosimetry allowed us to investigate the effect of hydrophobic porous materials on the shift of *T*_c_.

In refs (23−25,27), the authors reported that the critical temperature of a fluid confined in a pore shifts to lower values, the amount of shift depending on the pore size: the smaller the pore size, the lower the critical temperature (and critical density) of the fluid. Concerning simulations, Brovchenko et al.^31−33^ performed Gibbs Ensemble Monte Carlo simulations on TIP4P water in cylindrical pores,^31−33^ reporting a maximum reduction of the critical temperature of 40 K with respect to the bulk value for the water model used in the calculations. We speculate that the unprecedented results reported in our work are due to the confinement of the fluid within a hydrophobic MOF and other characteristics of the porous medium. Indeed, the reduction of the temperature induced by confinement has typically been studied in lyophilic/hydrophilic media, which allow capillary condensation from the bulk gas phase.

Thus, the observation of this exceptional decrease in the critical temperature within a class of largely overlooked systems sheds some light on the effect of hydrophobicity and topology of porous materials on the fundamental properties of confined fluids, further widening the technological application of heterogeneous lyophobic systems.

## 2 Results and Discussion

**Figure 1:**
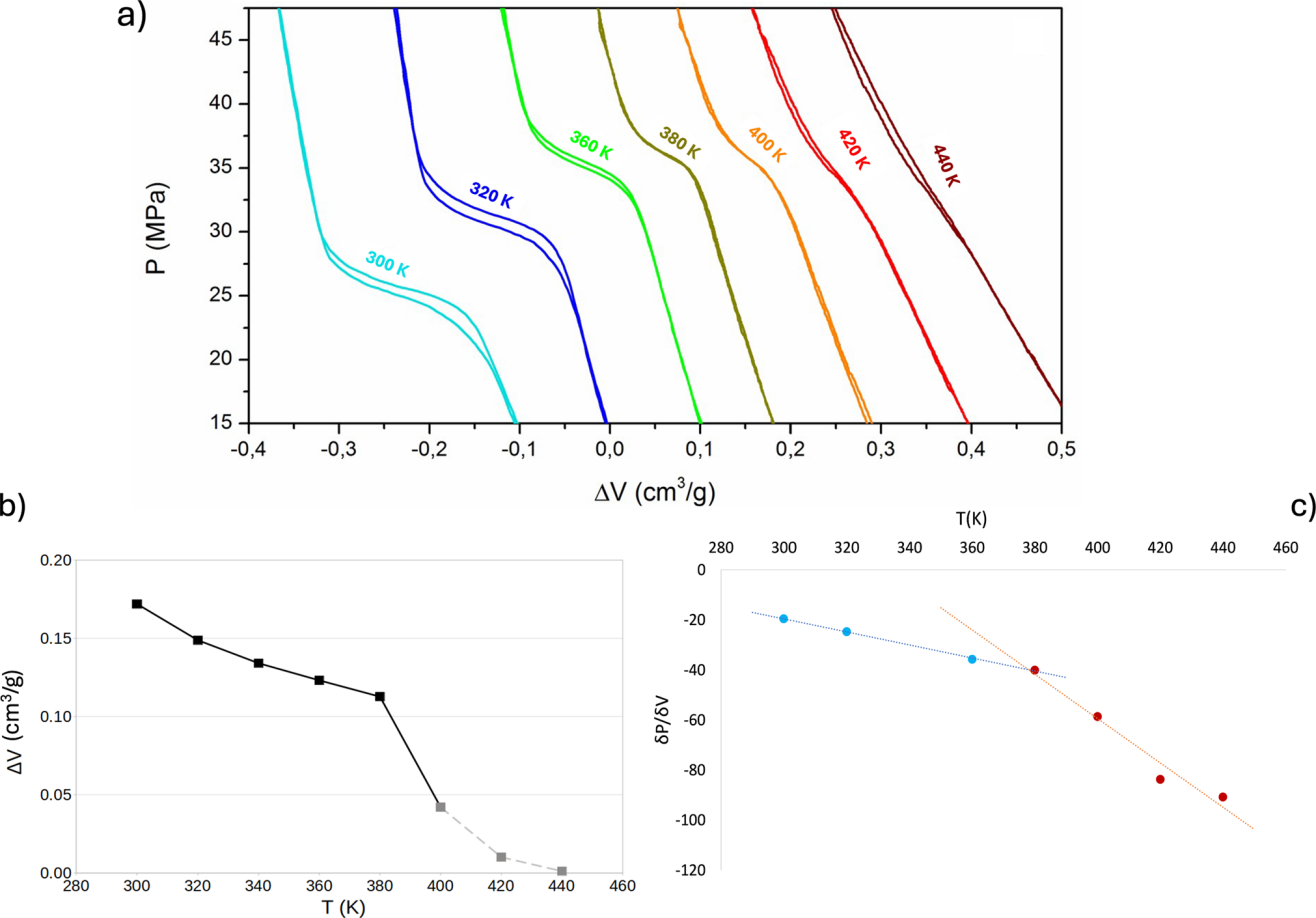
Intrusion/extrusion characteristics of {Cu_2_(tebpz) + water} as a function of temperature. (a) PV-isotherms at several temperatures. Δ*V* represents the change in the volume of the system. The value is negative, and upon intrusion, the overall volume of the system is reduced. PV-isotherms are shifted by 0.1 cm^3^/g along the Δ*V* axis to enhance readability, and intruded volume depends only on the length of the intrusion branch of the PV-isotherm, i.e., the plateau region of the curve, which does not change with the shift. A trend of the intrusion pressure with the temperature is observed. This phenomenon, which goes beyond the scope of this paper, is discussed in a forthcoming article. In panel (b), we report the intruded volume vs temperature curve, indicating the reduction of the intrusion volume as the temperature increases, characterized by a sudden drop at 380 K. In panel (c), we report the slope of the PV(*T*) curves vs *T*. At 380 K the drastic change of the trend is evident which represents the achievement of the supercritical state.

Cu_2_(tebpz) is a hydrophobic porous MOF^44,45^ characterized by an external surface contact angle of 123.6° at room temperature (Figure SI1). Its porosity consists of two channel-like apertures, the larger elliptical one of 0.67 × 1.3 nm^2^ size and the smaller circular one of 0.62 nm radius (Figure SI1). These cavities are connected by even narrower apertures on their lateral walls (secondary porosity). At ambient pressure, the porosity of the material is dry, with the channel possibly occupied by water in its confined vapor phase. However, upon the effect of sufficient hydrostatic pressure, the liquid can intrude into the material. The threshold pressure at which intrusion takes place is denoted as intrusion pressure. During intrusion, the overall volume of the liquid + porous solid sample reduces as some significant amount of water is transferred from the liquid bulk to the pores of the MOF. The experiment recording the volume of the overall sample corresponding to a given pressure for a prescribed temperature is dubbed liquid porosimetry, and the PV curves measured in these experiments are denominated PV-isotherms. Liquid porosimetry experiments were performed on the {Cu_2_(tebpz) + water} system to study the effect of the temperature (*T*) on the intrusion–extrusion process. Numerous cycles of intrusion/extrusion were made in a temperature range from 300 to 440 K, recording PV-isotherms (Figure 1a). It is known that intrusion–extrusion is accompanied by heat effects,^50^ and this in principle might perturbate isothermal conditions. In the reported experiments, this issue is addressed by performing intrusion–extrusion under quasistatic conditions by applying a very low compression–decompression rate (0.5 MPa/min). This ensures that the temperature is maintained constant during the compression–decompression cycle, as can be seen in Figure SI2. At lower temperatures, the isotherms present the characteristic discontinuity associated with the intrusion/extrusion process: the volume of the {Cu_2_(tebpz) + water} sample suddenly decreases/increases due to the displacement of the dense liquid inside/outside the material’s cavities, replacing/reinstating the vapor (gas) phase. This discontinuity shrinks with temperature, until disappearing at temperatures over 400 K. Here, the disappearance of PV-isotherms discontinuity is associated with the achievement of supercriticality following the methodology reported in the literature for hydrophilic porous materials, where condensation of vapors in their cavities (capillary condensation) is used instead of intrusion.^23,24,51−56^ Both in liquid intrusion/extrusion and in the capillary condensation, the disappearance of PV-isotherm discontinuity can be explained as follows. The change of the overall sample volume across intrusion (condensation), Δ*V*^intr^, is correlated to the difference between the densities of the liquid-like and the vapor-like confined fluid by the following relation:
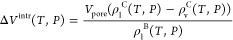
1where *V*_pore_ is the volume of the pores of the porous material, which for the sake of simplicity we consider independent of pressure and temperature. Here, ρ_l_^C^(*T*, *P*) and ρ_v_^C^(*T*, *P*) are the number densities of liquid-like and vapor-like confined fluid at temperature *T* and pressure *P*, respectively, and ρ_l_^B^(*T*, *P*) is the number density of bulk water. The numerator of eq 1 amounts to the number of water molecules entering the porous material (per gram of material) at the temperature *T* and pressure *P*, which, divided by the density of bulk water under the corresponding conditions, gives the volume of bulk liquid entering the pores. If ρ_l_^C^(*T*)−ρ_v_^C^(*T*) decreases, due to a decrease of the first term and an increase of the second, Δ*V*^intr^(*T*) decreases proportionally. When ρ_l_^C^(*T*) = ρ_v_^C^(*T*), i.e., when the confined fluid becomes supercritical, the intruded volume vanishes and so does the discontinuity in the isotherm. In intrusion/extrusion experiments, the vanishing of the intruded volume does not mean that the volume of the system along the isotherm does not depend on *P* because both the porous solid and bulk liquid can be compressed/expanded, and, especially, increasing/decreasing pressure continuously changes the density of the confined supercritical fluid.

In Figure 1b, we report the intruded volume vs temperature as obtained by the analysis of isotherms of Figure 1a. One sees a drastic reduction of intrusion volume to its near disappearance as temperature increases from 300 to 440 K. This is accompanied by a nonmonotonic change of the intrusion pressure, the origin of which will be discussed in a forthcoming article. We remark that the Cu_2_(tebpz) structure is preserved in the orthogonal *Pnnn* space group over the 278–428 K temperature range and for pressure between 0.4 and 35 MPa (Section SI1). In addition, no signs of the appearance of any new crystalline phase are observed. Over the above *P*–*T* range, the largest variation of lattice parameters is 1.6%, which clearly cannot account for the dramatic reduction of the intrusion volume shown in Figure 1b. These findings are consistent with previous literature data reported by some of the authors.^20^ Additionally, intrusion/extrusion can only be partially reversible, i.e., after a complete cycle, water may remain inside the MOF pores. Here (Figure 1), the analysis is performed on the intrusion/extrusion cycle after the first one has been completed; hence, the phenomena discussed here and in the following are genuine and repeatable once and not transient events related to the achievement of the stationary condition of the fluid inside the porosities (see Figure SI3).

Summarizing, the sudden and large reduction of the intrusion volume cannot be ascribed to any structural change of the MOF caused either by the growing temperature or growing pressure or combination of both.

A complementary analysis of the PV-isotherms consists of computing the slope of the intrusion/extrusion plateau. Intrusion (extrusion) amounts to a confined phase transition, condensation (evaporation). In the bulk, condensation (evaporation), corresponding to the intrusion section of the PV-isotherm, would be linear with a zero slope. Under confinement, one observes some slope because of the polydispersity of the sample. Even in the case of porous crystalline materials, such as MOFs, there is a dispersion in the crystallite size, and we have recently shown that this affects the intrusion pressure,^57^ resulting in the slope of the intrusion/extrusion plateau. As noted by Morishige and Ito for capillary condensation,^47^ this slope significantly grows when the confined fluid becomes supercritical.

This analysis has been extensively used to investigate the supercritical transition in confined fluids, providing a solid methodological background to our investigation approach.^25,29,36,47,52,56,58−62^ At the same time, to the best of our knowledge, this is the first time this approach has been applied to hydrophobic (or, more in general, lyophobic) porous media, which require the use of liquid porosimetry to obtain the formation of a confined liquid phase. The combination of a well-established methodology and an alternative approach allowed us to discover the exceptional shift of *T*_c_ discussed in the following.

In Figure 1c, one notices a sudden change of the slope of the intrusion branch of PV-isotherms at 380 K, further supporting the hypothesis derived from the trend of Δ*V*^intr^ versus *T* that above this temperature the system has become supercritical.

Concerning the trend of the intruded volume with the temperature, another possible explanation of its sudden and marked reduction at higher *T* is that at this temperature intrusion pressure is too high, beyond our experimental limit. To test which hypothesis, whether supercriticality or liquid extrusion, is more likely to explain the experimental results, and to characterize the effect of temperature on intrusion/extrusion at an atomistic level, we performed molecular dynamics simulations. We considered a 4 × 1 × 1 slab of Cu_2_(tebpz) between two thick films of water. Additional computational details are provided in Section 4. Experimentally, at 300 K, intrusion (and extrusion) takes place between 22 and 27 MPa (Figure 1a). In the simulations, it is possible to keep the system in its initial, metastable, extruded state at even higher pressures (see refs (1−3) for more details about intruded/extruded metastable states). This is because the intruded and extruded states are separated by a free energy barrier that prevents intrusion and/or extrusion on the nanosecond time scale of the simulations. These metastabilities make it possible to independently investigate the effect of thermodynamic conditions on the confined vapor and liquid water phases, which will help us to assess the origin of the complex experimental phenomenology.

The intruded phase is generated by first applying a large, 200 MPa, pressure to force water to enter in the MOF on the short nanosecond time scale of the simulations and then relaxing the system to the in silico operative conditions discussed in the following. This large pressure prevents us from investigating the realistic intrusion path, as we have done in the past for other hydrophobic MOFs.^1,2,4,63−65^ However, this is not the objective of this work, and the procedure highlighted above is computationally more efficient (Section SI2).

**Figure 2:**
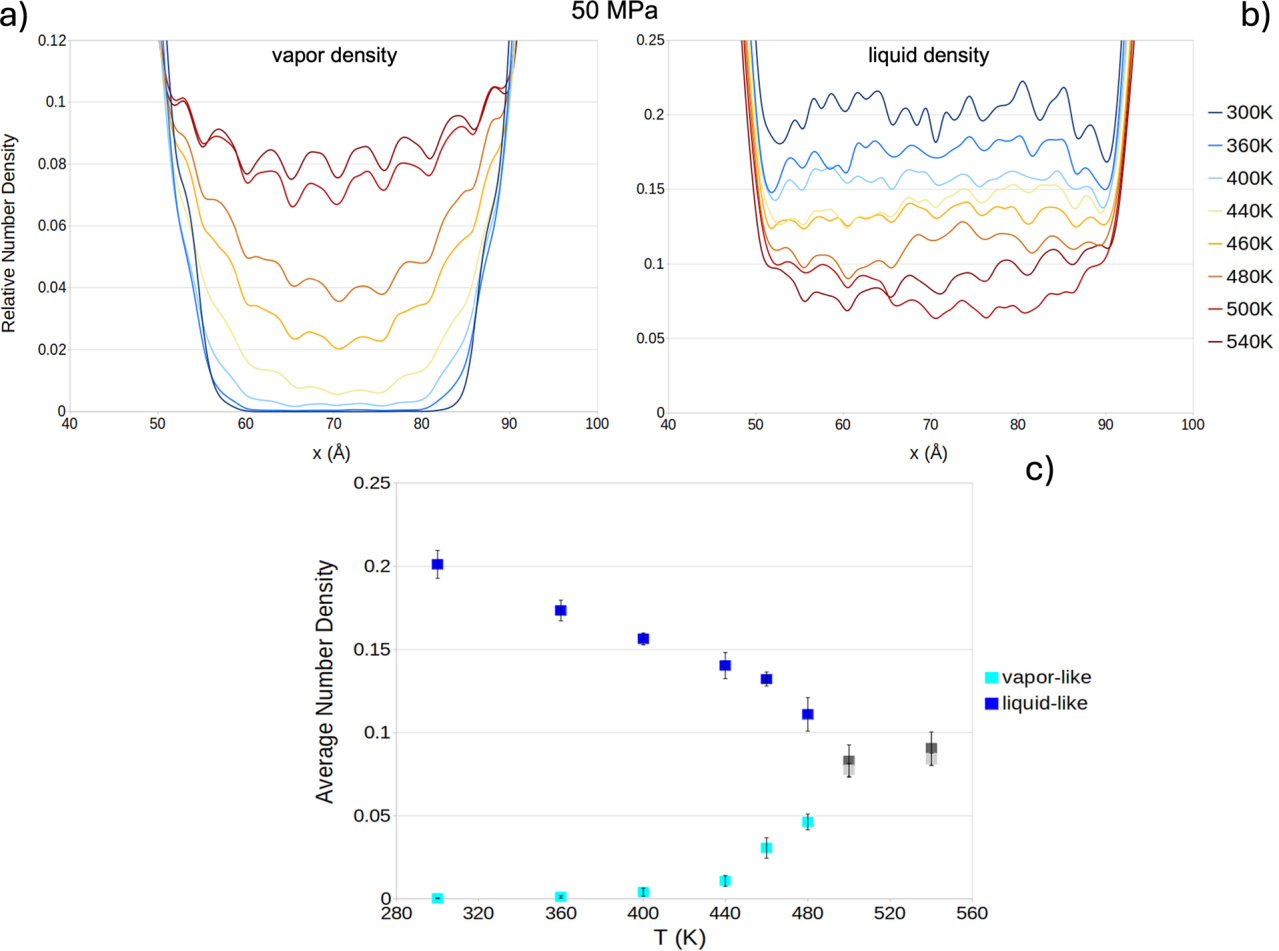
(a) Profiles of vapor-like and liquid-like density in the 300–540 K temperature range at 50 MPa, normalized with respect to bulk water. (b) Normalized average density of liquid-like and vapor-like water vs temperature at 50 MPa. Coincidence between the density of the putative liquid-like and vapor-like phase at 500 K suggests achievement of the supercritical phase of confined TIP4P/2005 water, 140 K down from the 640 K bulk value.

The characteristics of the intruded system are studied at 50 MPa over the broad 300–540 K temperature range. Pressure is set higher than the maximum considered in the experiments, and the temperature range is broader to allow for possible imperfections in interatomic force models. While some quantitative mismatch with experiments is observed (see below), the force model employed in this work correctly reproduces the qualitative characteristics of the complex HLS (Section SI2). The quantitative mismatch is not surprising considering that neither the liquid nor the interface solid/liquid force model has been optimized to represent water under the extreme confined state imposed by the Cu_2_(tebpz) MOF. Indeed, despite the relatively simple force model, the agreement between experimental and theoretical results is surprisingly good. Consistently with synchrotron data (see below), apart from a few molecules quickly passing through circular channels (Section SI3), in both the vapor-like and liquid-like states, water is found only in the elliptic pores of Cu_2_(tebpz). We computed the relative (to bulk water) density profile of confined liquid-like (Figure 2a) and vapor-like (Figure 2b) water at several temperatures in the range of 300–540 K. Here and in the following, we focus more on the trend of the density of the confined phases, rather than on their absolute value. Indeed, for the latter, one needs a careful evaluation of the volume occupied by the fluid, and that on the nanoscale of the Cu_2_(tebpz) pores is estimated with limited accuracy. Density profiles present alternating maxima and minima due to the complex shape of the MOF’s cavities (Figure SI13). This aspect is discussed in more detail in the following section. Figure 2c presents the average relative density of the liquid-like and vapor-like water in the elliptical channels.

**Figure 3:**
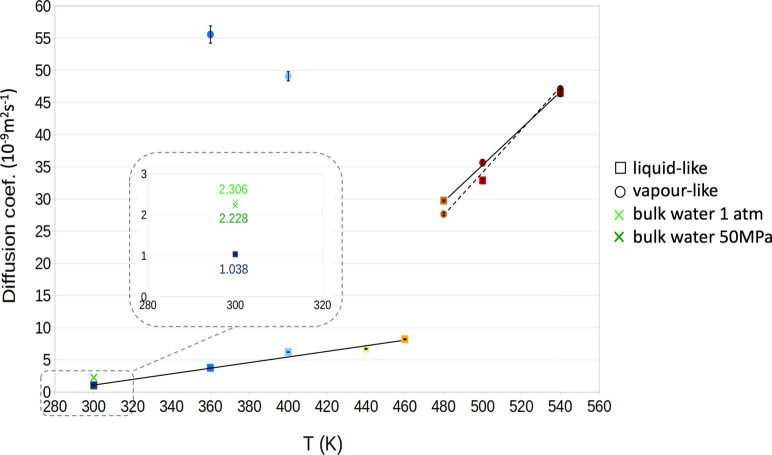
Diffusion coefficient of bulk and confined water as a function of temperature. For water, data are reported at 1 atm and 50 MPa. For the confined phases, data are reported at 50 MPa.

Figure 2 shows a decrease in the density of the liquid-like phase and an increase in the density of the vapor-like one. This is consistent with the typical effect of temperature on the densities of liquids and vapors. In the vapor-like phase, the change of density becomes more marked at ∼460 K. A qualitatively similar trend is observed in the bulk, where, especially for the vapor phase, the water vapor density changes more markedly starting from ∼50 K before the critical point. At 500 K, the densities of confined water of the initially intruded and extruded systems are indistinguishable, indicating that the system has reached the confined critical conditions, ∼140 K lower than the bulk value of TIP4P/2005 (Figure 2c). To assess whether this trend of water density in Cu_2_(tebpz) channels is related to a supercritical transition rather than either a temperature-induced intrusion or a condensation, we investigated the trend of the (self-)diffusion coefficient with *T* in the liquid-like and vapor-like phases. One notices that the diffusion coefficient of the liquid-like phase initially grows smoothly with temperature, showing a marked discontinuity at 480 K, i.e., when the system approaches the putative confined critical temperature (Figure 3). The estimation of the diffusion coefficient of the confined vapor-like water is more difficult to obtain with sufficient accuracy given the low density of this phase. Nevertheless, we were able to obtain reliable data for values in the desired region across the supposed confined critical transition. One notices that before the putative critical temperature, the diffusion coefficient of the gas phase decreases with *T* due to the marked increase of the density with temperature. For temperatures higher than the presumed confined *T*_c_ the difference between the diffusion coefficients of the initially vapor-like and liquid-like states is very small and for *T* = 540 K within the statistical error, confirming that confined water does not support distinct vapor-like and liquid-like states any longer, i.e., confined water is supercritical. This conclusion is reinforced by the observation of the peculiar trend of the diffusion coefficient of the two confined phases, the discontinuity across the apparent supercritical transition for liquidlike water, and the inversion of the trend with *T* for the vapor-like one. A complete characterization of confined supercritical water requires an analysis of other properties, e.g., heat capacity, the existence of a confined pseudocritical temperature, etc. However, this goes beyond the scope of the present work, which is focused on the finding of an unknown intrusion/extrusion phenomenology. Thus, a complete characterization of the putative critical transition in water confined within Cu_2_(tebpz) and other hydrophobic porous media is left for a forthcoming work.

Consistently with eq 1, our simulations suggest that the disappearance of the intrusion process at 400–440 K observed in the experiments is due to a ∼200 K reduction of the critical temperature of real water due to its confinement within Cu_2_(tebpz) pores. Indeed, the fact that confinement can reduce the critical temperature has been previously discussed in the literature,^12,23,25,36,52−54,61,62,66,67^ but theoretical estimations of the reduction of the critical temperature are much lower^31−33^ (Δ*T*_c_ < 100 K) and no experimental evidence of such large *T*_c_ downshift is available.

**Figure 4:**
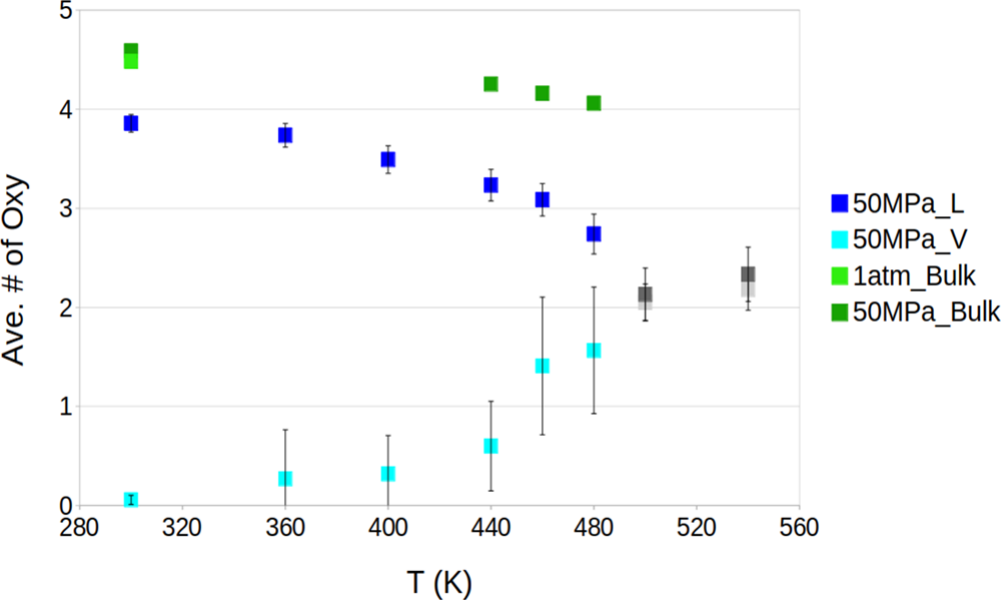
Average number of nearest neighbor oxygen atoms for liquid-like and vapor-like confined water molecules in the 300–540 K temperature range. Data for bulk water are also reported.

**Figure 5:**
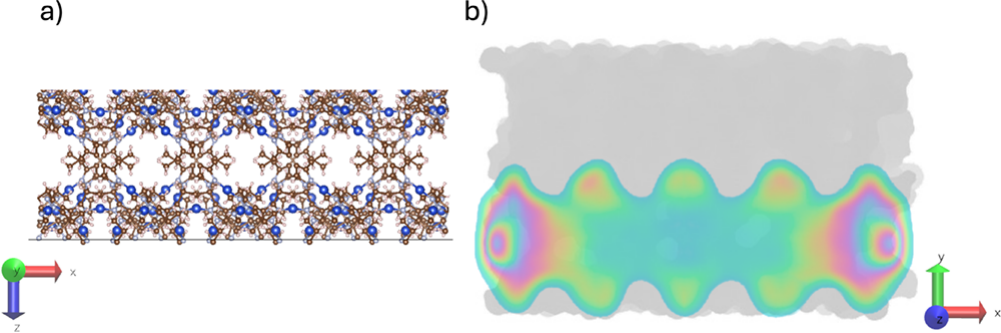
(a) Secondary porosity of an elliptical channel of Cu_2_(tebpz) consisting of small lateral pores. (b) *xy* plane density map plane of oxygen atoms of vapor-like water molecules obtained from 10 ns MD run at 440 K and 50 MPa. Focusing on the inner part of the channels, one observes a higher density of oxygen atoms in the secondary pores on the lateral walls of the elliptical channels. Of course, a high water density is also observed at the two opposite entrances of the channel, but this is due to the protruding liquid meniscus. In the water density map, green denotes low density, and yellow to purple denotes higher density.

Summarizing, a comparison between experimental intrusion volume and computational density vs *T* suggests that the change and, finally, the disappearance of the latter are due to the large ∼200 K critical temperature downshift for water confined within the narrow pores of Cu_2_(tebpz). This is further confirmed by the sudden, more marked decrease in the intrusion volume in a 50 K temperature range before the disappearance of any intrusion. To the best of our knowledge, this is the first experimental evidence, supported by computational results, of such a large reduction of the critical conditions due to confinement. The shifting of the critical temperature in the present work and the literature can be (and was) attributed to the reduction of the number of hydrogen bonds induced by confinement.^21,22,26,30−32,41,43^ To assess whether the origin of the drastic reduction of *T*_c_ reported in this work has the same origin, we computed the number of nearest neighbors of liquid-like and vapor-like water confined within Cu_2_(tebpz) (Figure 4). For the liquid-like case, we compare results with data from genuine bulk water determined under the same thermodynamic conditions. As expected, at the low temperature, the number of nearest neighbors of confined water is already lower than that of the bulk liquid although this difference is limited. This difference grows at higher temperatures, with the difference of coordination number between bulk and confined water reaching the value of 1 at 480 K, the last temperature in our simulations where we distinguish two confined phases: the number of nearest neighbors is ∼3 for confined water versus ∼4 for its bulk counterpart. These differences between bulk and confined liquid water are analogous to literature results on cylindrical channel systems,^31−33^ suggesting that the origin of a much higher reduction of the critical temperature for water confined in Cu_2_(tebpz) may reside in the properties of the gas-like phase. Indeed, for this latter phase, we observe a large value of the number of nearest neighbors, ∼2, already at 460 K. Such a large value of nearest neighbor led us to speculate that at high temperatures the gas-like phase consists of small, trimer, water clusters rather than independent molecules, with each molecule of the cluster forming hydrogen bonds with the other two in a cyclic structure (Figure SI16). Indeed, the formation of a water cluster was also postulated for water confined in narrow (2–3 nm) carbon nanotubes even under ambient conditions.^68^ Moreover, water trimers (and dimers) are one of the typical structures present in supercritical bulk water.^60,69^ We believe that this characteristic of the gas-like phase is responsible for the large downshift of the critical temperature. To the presence of such clusters must correspond a high local density of the vapor-like phase. Indeed, the density profile of the vapor-like phase (Figure 2a) at higher, confined subcritical temperatures presents maxima. These maxima are in correspondence with lateral apertures of the elliptical channels, as confirmed by the color map of the water density shown in Figure 5. Such a water density should be reflected in an excess electronic density, with respect to the case of Cu_2_(tebpz) in the air. This excess density should be localized near the lateral apertures, at a short distance from Cu atoms. To validate this hypothesis we performed synchrotron in situ powder diffraction measurements at 17-BM beamline at Advance Photon Source at 363.15 K up to 35 MPa, which is below the liquid intrusion pressure of Cu_2_(tebpz), and only vapor is expected within the pores (Section SI1). Indeed, synchrotron data shows that under such conditions of pressure and temperature, there is (i) a significant amount of vapor in the center of the MOF elliptical channels and (ii) additional water molecules are located at the apertures on the lateral MOF’s walls, at a ∼0.2 nm distance from copper atoms. This distance is similar to the 0.22 nm reported earlier for another Cu-based MOF (HKUST-1),^70^ containing up to 1 water molecule per copper atom in the room temperature structure. These synchrotron results are consistent with molecular dynamics data. Indeed, we observed a large vapor density in the cavities as the pressure and temperature increased, with maxima of the water vapor density field at ∼0.29 nm from Cu. The quantitative mismatch between the Cu–H_2_O distance determined from synchrotron measurements and the maximum of the water density field (Figure 5b) as measured by molecular dynamics^71,72^ is due to the fact that in the former one determines only the distance of molecules with a relatively long residence time, which are expected to be at shorter distances from copper atoms. Therefore, as we anticipated in Section 1, the copper atoms in the channels appear to represent locally attractive sites for water molecules despite the general hydrophobicity of the entire solid.

### 2.1 Case of ZIF-8

To show that the phenomenology and conclusions drawn so far for Cu_2_(tebpz) are not limited to this MOF, we also considered the widely studied hydrophobic crystalline porous material, ZIF-8^18,73−75^ (Section SI4). ZIF-8 is another relatively thermally stable MOF, which allowed us to investigate its intrusion–extrusion characteristics over a broad temperature range. Water intruded in ZIF-8 shows strong analogies with that intruded in Cu_2_(tebpz). In liquid porosimetry experiments, we observe a marked reduction of the liquid density as temperature increases (Figure SI18). However, the fingerprint of a supercritical transition, namely, the disappearance of intrusion altogether, is not seen due to the limited temperature range that can be investigated on account of the degradation of this MOF above ∼360 K. The same rationale does not hold in simulations as one can prevent ZIF-8 degradation even at very large temperatures. Here, we observe a trend of the density of the liquid-like and vapor-like phase similar than with Cu_2_(tebpz), which led us to predict a computational supercritical transition at ∼500 K (Figure SI19).

## 3 Conclusions

In this work, we report unexpected properties of water intruding into microporous hydrophobic MOFs, such as Cu_2_(tebpz) and ZIF-8. In particular, simulations have shown that the peculiar trend of PV-isotherms with temperature in Cu_2_(tebpz) is to be ascribed to an unprecedented critical temperature reduction of 200–250 K. This achievement was enabled by the exploration of confined fluids under liquid intrusion conditions, instead of widely considered capillary condensation. Liquid intrusion allows us to explore (i) hydro(lyo)phobic systems and (ii) pressures higher than the critical value of water, which is impossible with capillary condensation. For ZIF-8, whose thermal stability is limited, it was impossible to reach a high enough temperature to observe a confined supercritical transition. Nevertheless, both the experimental trend of the intruded volume with temperature and computational results, which could reach higher temperatures, suggest that also this MOF is able to significantly decrease the water *T*_c_. Present results highlight the importance of developing/fabricating thermally stable MOFs and, in general, porous systems to thoroughly explore hydrophobic confinement on critical shifting. Interestingly, present experimental and theoretical results are at odds with previous theoretical works,^33^ predicting that large *T*_c_ reduction requires highly hydrophilic solids. Apparently, the large reduction of the critical temperature is not mainly due to the decreased number of water–water hydrogen bonds in the liquid-like phase under high confinement but to the large density of the vapor-like phase. This hypothesis is confirmed by synchrotron measurements. Here, at high temperatures, water clusters, possibly trimers, appear. We believe that the formation of such clusters is promoted by the peculiar geometry and chemistry of Cu_2_(tebpz), with some of the Cu atoms of the framework relatively accessible to water molecules. Indeed, MDs, liquid porosimetry, and synchrotron results suggest that despite an overall hydrophobicity of the MOF, there are locally attractive interactions that determine its properties, such as the observed large reduction of the critical temperature. Also in this case, this phenomenon was not considered in the previous literature in the field, where the focus was mostly on geometrically simpler porous media. Present findings allow us to envisage novel technological applications of hydrophobic porous media characterized by microscopic pores. For example,^34,79^ considering the increase of the heat capacity in the proximity of the critical conditions, one might exploit high water confinement to achieve efficient thermal energy storage with supercritical water at more moderate thermodynamic conditions of ∼35 MPa and ∼400 K in the case of {Cu_2_(tebpz) + water} system. In this regard, we believe that a systematic investigation of a wide range of porous materials is needed, considering the chemical and physical characteristics of heterogeneous lyophobic systems, and we hope that this will promote the contribution of the research community active in the field.

## 4 Experimental Section

The Cu_2_(tebpz) MOF with 1D nanoscale channels featuring hydrophobic ethyl groups was synthesized according to the procedure described by Wang et al.^44^ Distilled water was used for intrusion–extrusion tests. Transitiometer^76,77^ from BGR-Tech was used for intrusion–extrusion experiments recording PV-isotherms at various temperatures. The compression rate of 0.5 MPa/min was used for the presented experiments. Experiments were performed following the protocol described elsewhere.^50^ Stability of Cu_2_(tebpz) MOF after intrusion–extrusion cycling at various temperatures was previously confirmed.^20^ Details regarding calorimetry and structural (PXRD) analysis are reported in the Supporting Information.

### 4.1 Computational Details

Classical Molecular Dynamics simulations were performed using LAMMPS.^78^ The water molecules were represented by the TIP4P/2005 model.^79^ The force field for the MOF were generated using UFF4MOF,^80^ while the partial charges of the atoms were calculated by ab initio methods (Bader charges/Lowdin charges comparison) with QUANTUM ESPRESSO.^81^ Cu_2_(tebpz) interacts with H_2_O via electrostatics plus the modified Lennard-Jones interaction to tune the hydrophobicity of the MOF. The computational sample consists of a 4-unit-cell-thick slab of Cu_2_(tebpz) (∼4.2 nm) and ∼2000 water molecules for a total (Cu_2_(tebpz) + H_2_O) of ∼10,700 atoms. Periodic boundary conditions were applied along the b, c lattice directions, while a pair of pistons were introduced to control the pressure applied to the liquid along the a direction, which is the same direction in which the MOF unit-cell is replicated 4-fold. This approach was successfully implemented on other heterogeneous systems^18,64^ and implements the prescriptions for heterogeneous systems introduced by Marchio et al.^82^ SASA and MOF solvent accessible volume to determine, for example, liquid and vapor densities were estimated using ZEO++.^83−85^ MD intrusion/extrusion experiments were performed within the constant number of particles, constant pressure, and constant temperature (NPT) at several values of pressure (25, 35, 45, 50, 200 MPa) and regular temperature intervals in the range 300–540 K. In particular, the intruded system was obtained first by applying a large pressure of 200 MPa (Brute force approach), to obtain water intrusion on a relatively short computational time, and then released to the target thermodynamic conditions by running extended simulations until stationarity is reached (5 ns for each relaxing step and 10 ns for stationarity), including stationarity of the water density field within Cu_2_(tebpz) channels.

## Supporting Information Available

Additional experimental and computational details and methods (PDF)
