# Supplementary material for: Mild-Temperature Supercritical Water Confined in Hydrophobic Metal–Organic Frameworks
Source: J Am Chem Soc. 2024 May 3;146(19):13236–46. doi: 10.1021/jacs.4c01226 (PMC11099966; doi:10.1021/jacs.4c01226)
Supplement: Supplementary file 1 — ja4c01226_si_001.pdf [file ja4c01226_si_001.pdf]

Supporting Information

Mild-temperature supercritical water confined in hydrophobic metal-organic frameworks

Sebastiano Merchiori[1], Andrea Le Donne[1*], Josh D. Littlefair[1], Alexander Rowland Lowe[2], Jiang-Jing Yu[3], Xu-Dong Wu[3], Mian Li[3], Dan Li[4], Monika Geppert-Rybczynska[2], Lukasz Scheller[5], Benjamin A. Trump[7], Andrey A. Yakovienko[8], Paweł Zajdel[5*], Mirosław Chorążewski[2*], Yaroslav Grosu[2,6*], Simone Meloni[1]

[1] Department of Chemical, Pharmaceutical and Agricultural Sciences, University of Ferrara, 44121 Ferrara, Italy.

[2] Institute of Chemistry, University of Silesia, Szkolna 9, 40-006 Katowice, Poland.

[3] College of Chemistry and Chemical Engineering, Shantou University, and Chemistry and Chemical Engineering Guangdong Laboratory, Guangdong 515063, China.

[4] College of Chemistry and Materials Science, Jinan University, Guangzhou 510632, China.

[5] Institute of Physics, University of Silesia, 41-500 Chorzów, Poland.

[6] Centre for Cooperative Research on Alternative Energies (CIC energiGUNE), Basque Research and Technology Alliance (BRTA), 01510 Vitoria-Gasteiz, Spain.

[7] NIST Center for Neutron Research, National Institute of Standards and Technology, Gaithersburg, Maryland 20899, USA.

[8] X-Ray Science Division, Advanced Photon Source, Argonne National Laboratory, Argonne, Illinois 60439, USA.

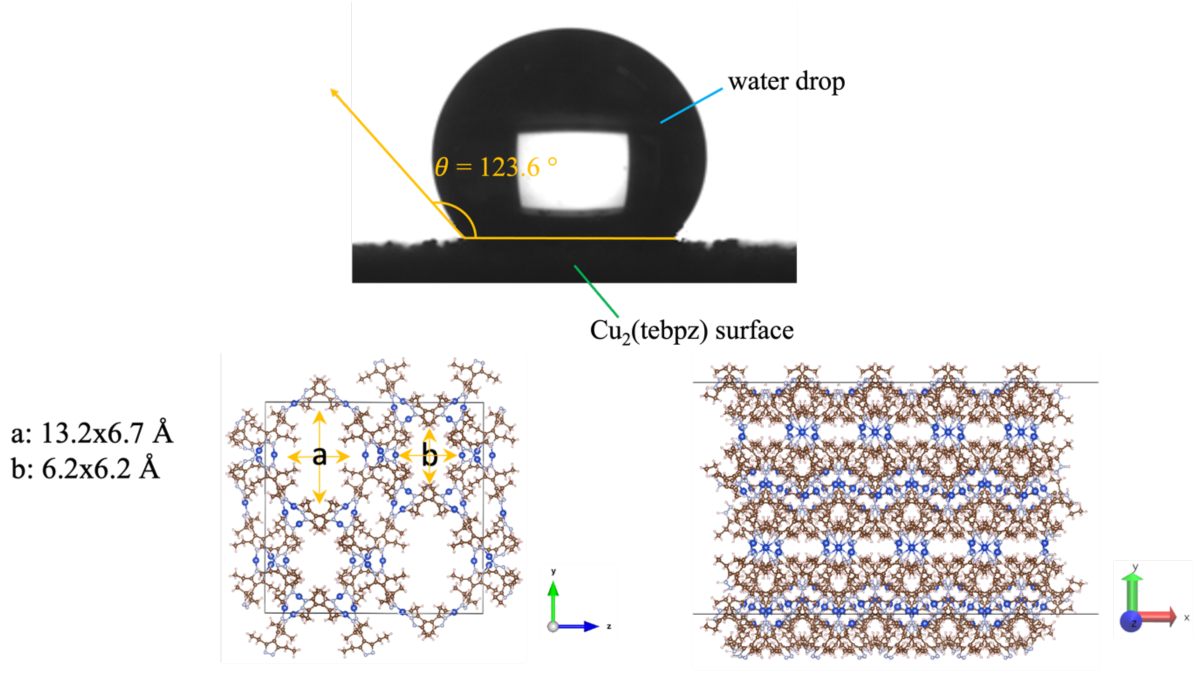

Figure SI1. Experimental contact angle (θ = 123.6°) measured at room temperature and structure of Cu_2(tebpz). This MOF is essentially characterized by two types of channels with elliptical and circular apertures running along the main axis of the solid. The elliptical channel (a) consists of an aperture of 1.32x0.67 nm^2 size, while the circular channel consists of an aperture of radius 0.62 nm. These cavities are connected both laterally and by even narrower openings in the walls, representing secondary porosity.

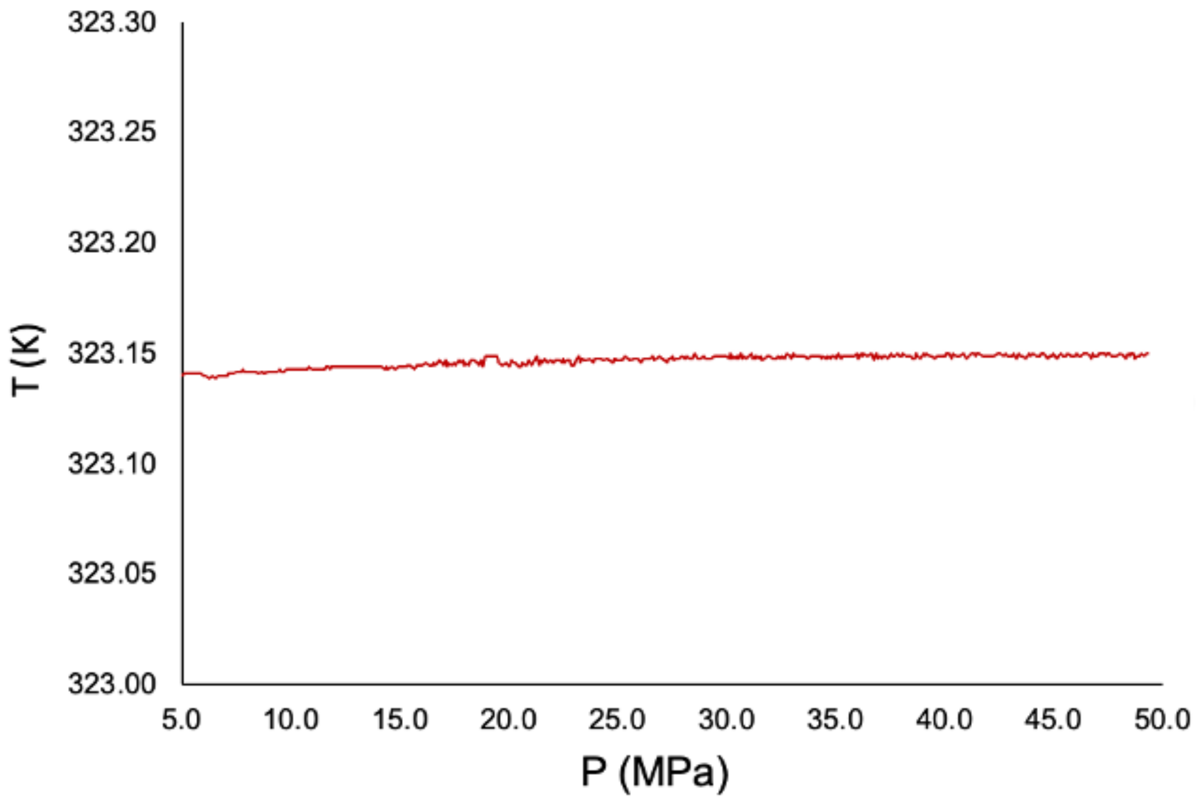

Figure SI2. Temperature trend in function of pressure during intrusion/extrusion cycle at the set temperature of 323.15 K. As can be seen, the slight non-linearity of temperature at low initial pressures is due to the compressibility of the liquid, which has a subtle effect on stationarity. However, once the intrusion and post-intrusion pressures are reached, a quasi-static condition is obtained. This condition has been verified and experimentally validated for each measurement.

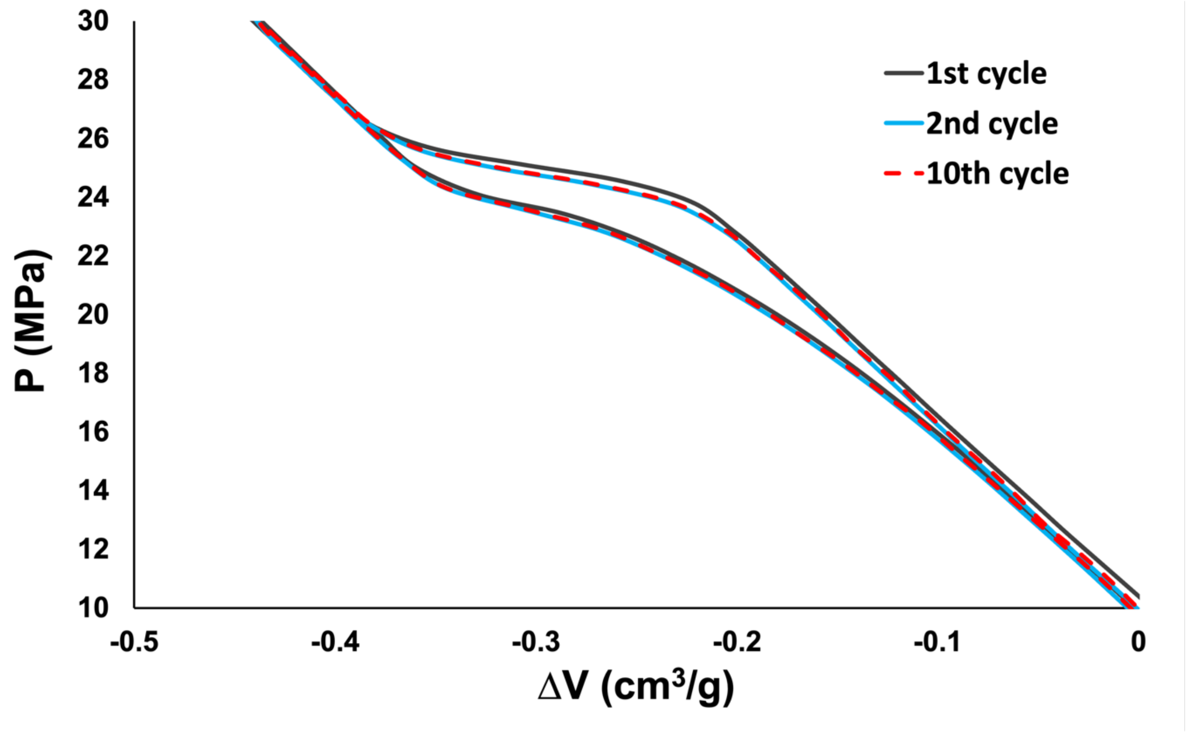

Figure SI3. PV-isotherms related to the first, second and tenth cycles of intrusion/extrusion of water in Cu_2(tebpz). As can be seen there is no significant difference between the first and the following cycles.

Section SI1 - Structural studies using *in-situ* powder diffraction.

A powder diffraction study of intruded and extruded Cu_2(tebpz) was carried out at the 17BM beamline of the APS, Argonne, USA. Real time intrusion/extrusion has also been studied but this is of no relevance for the subject of this research. This investigation aims i) to show the stability of the material under operative conditions, to support the claim that the significant shrink of the intruded volume is not due to a structural modification with applied pressure and/or temperature. Additionally, ii) these synchrotron data support the computational findings of the accumulation of water vapor in the extruded phase at the apertures on the lateral, inner walls of the MOF, namely in correspondence of the copper atoms.

Measurements were carried out with a beam of wavelength λ = 0.45181 Å. Concerning the sample, a specimen was loaded into a sapphire capillary. Temperature was measured by a K-type thermocouple placed inside the powder, paying attention that this was outside the region of the beam. The temperature was stabilized using a Cryostream 500+ controler. The pressure was dynamically stabilized using an ISCO syringe pump. Data were collected using a 2D detector and integrated using the GSAS-II package. Profile refinement was carried out using Fullprof Suite in line with the procedure used eariler in a similar study.[1]

Figure SI4 illustrates full profile refinement of a pattern collected at P = 0.4 MPa and T = 278.15 K, where the MOF, suspended in water, is in its extruded state. There is a good agreement between the model and the diffractogram. The refined lattice parameters in the orthorhombic space group *Pnnn* were (in Å) a = 10.2653(5), b = 32.647(2), c = 33.466(2). We remark that we retained the original atomistic structure of Wang et al.[2] submitted to the CCDC with identifyee #992002. We assume this structure to be correct in terms of topology, connectivity, and linker positions, though possible disorder of ethyl groups has not been taken into account in the original work.

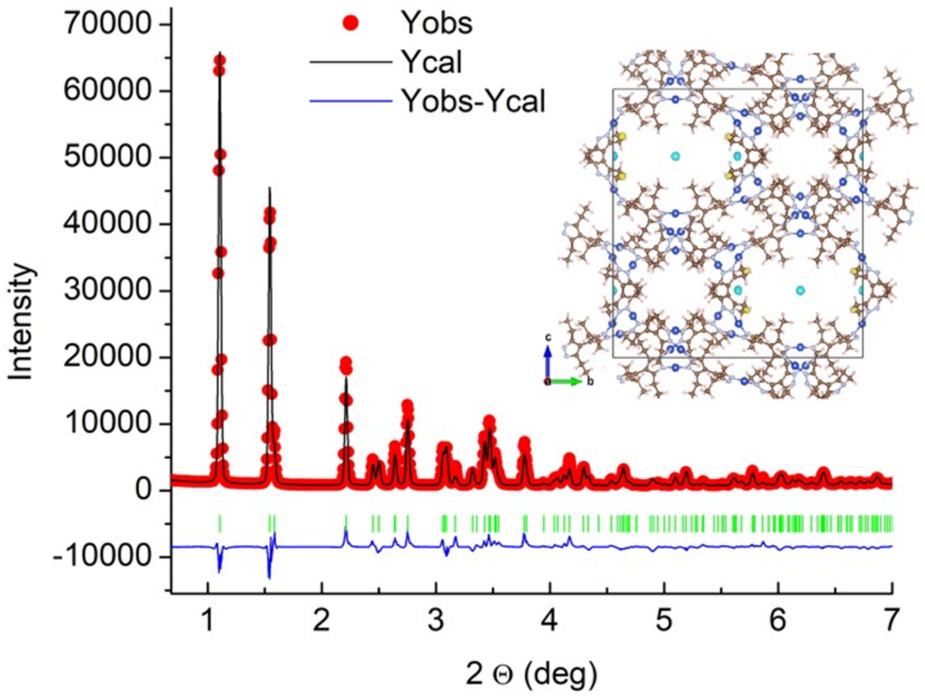

Figure SI4. Full profile refinement of Cu_2(tebpz) at 0.4 MPa and 278.15 K, before any intrusion/extrusion cycle. Red dots – measured intensities, black line – fit, blue line – difference between fit and experiment, green markers – Bragg peak positions. Inset presents difference synthesis at isosurface level 0.11 q/Å^3.

Figure SI5 presents synchrotron data for Cu_2(tebpz) at 35 MPa and 363.15 K. The Le Bail fit, requiring no information about the atomistic structure, shows no emergence of new peaks with respect to the extruded case at ambient pressure and 278.15 K. This indicates that the *Pnnn* symmetry of the MOF structure is preserved, and no extra phases is present in the sample. The Le Bail fit also provide lattice parameters at these thermodynamic conditions (in Å): a = 10.2591(1), b = 32.6257(5), c = 33.2268(4). Though changes of the lattice parameter upon the large increase of pressure and and temperature are observed, their magnitude is limited, ~ 0.02 Å along a and b and ~ 0.2 Å along c, amounting to a change between 0.06 and 0.6% of the lattice parameters.

A full profile refinement at the fixed atomistic coordinates of the deposited structure, and without taking into account water contained within the MOF, is performed on data in Figure SI6. From this, one can obtain a *Fourier difference*, a difference in the electronic structure between the present and the reference structure. This reveals a significant excess of electronic density with respect to the empty structure at the center of elliptical pores and in correspondence of the lateral apertures of the MOF's walls, in proximity of copper atoms. We attribute this excess of electronic density with respect to the reference structure to vapor-like water within the MOF's cavities. This is in remarkable agreement with the computational results, according to which at high pressure and temperature there is a dense vapor in the Cu_2(tebpz) elliptical channels. Moreover, this vapor is located in the positions predicted by simulations.

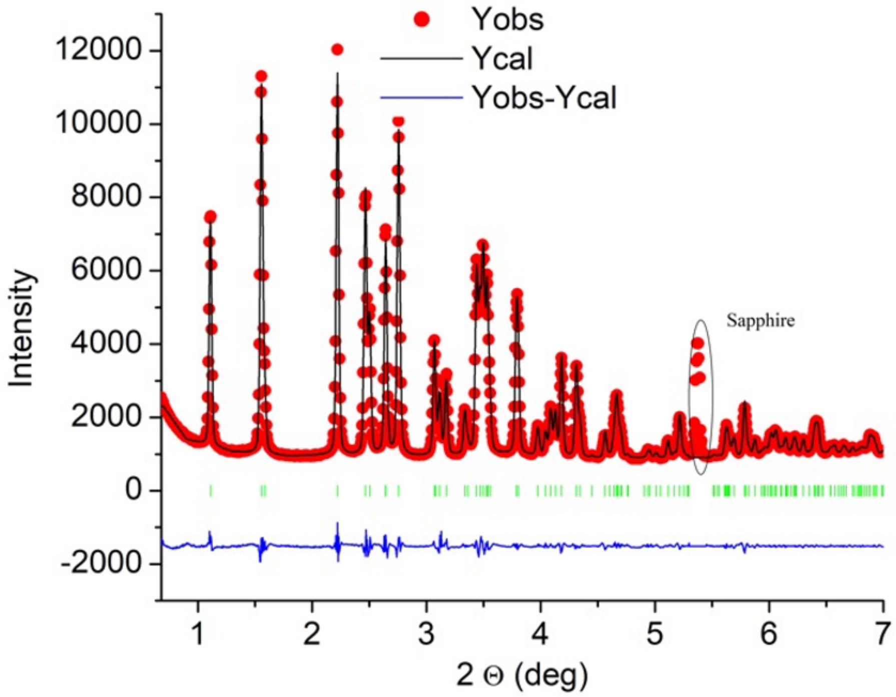

Figure SI5. Le Bail fit Cu_2(tebpz) at 35 MPa and 363.15 K in the extruded state. At 363.15 K the system is at the maximum pressure before intrusion. A reflection from the single crystal sapphire capillary is seen (marked with the oval).

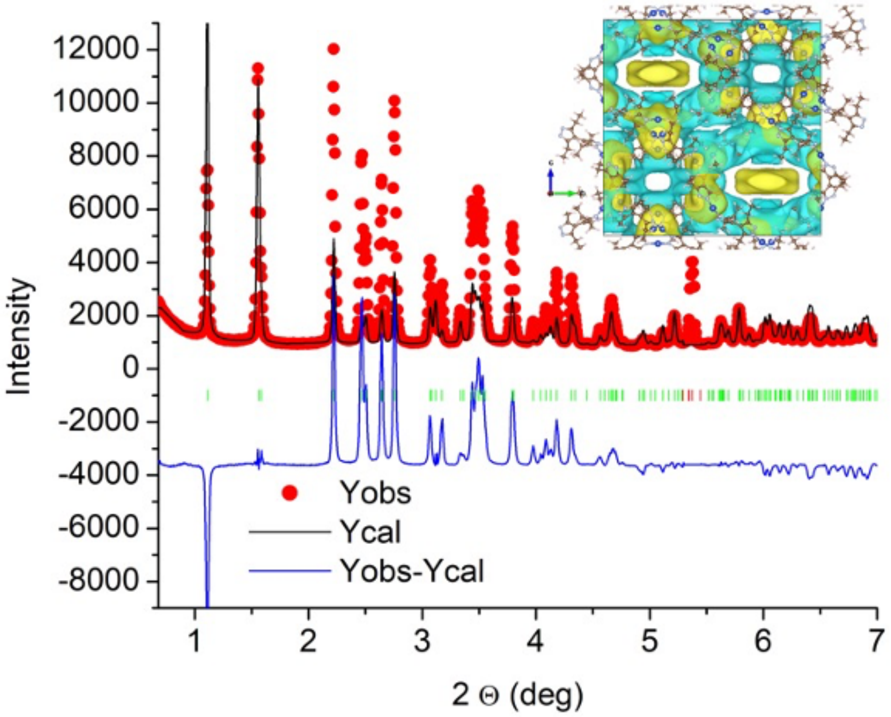

Figure SI6. Full profile refinement (fixed MOF coordinates and without including water in the fitting) of the intruded Cu_2(tebpz) at 35 MPa and 363.15 K. Inset presents residual density (yellow excess, blue defect) at 0.11 q/Å^3 isosurface level using the empty structure as the reference.

Figure SI7 presents Rietveld fit (fixed coordinates) of Cu_2(tebpz) at 0.4 MPa and 278.15 K after an intrusion/extrusion cycle. In other words, Figure SI7 is the same as Figure SI4 at the end of an intrusion/extrusion cycle. The refined lattice parameters are (in Å): a = 10.2148(7), b = 32.609(3), c = 33.325(2). The differences between the values refined from this fit and the one at the beginning of the experiment come most likely from the remnant water left in the pores (*vide infra*). The *Fourier difference* with respect to the case before intrusion reveals an excess of electronic density in the MOF, which is attributed to the presence of remnant water left in the pores. Remarkably, this excess of water is located in the proximity of lateral apertures, where excess density was found in the extruded state at higher temperatures/pressures and in simulations. In particular, the maximum of this excess density is at ~ 2 Å from the Cu sites (Figure SI8), which is comparable to the distance reported in another Cu-based MOF, HKUST-1,[3] for which structural water is reported. Once again, these findings are consistent with molecular dynamics data for the vapor-like phase, supporting the conclusion that the high density of water in the cavities is due to the strong H_2O-Cu interactions. As already mentioned in the main text, the mismatch between the predicted Cu-H_2O distance between synchrotron and molecular dynamics is mainly attributed to the different nature of water that can be appreciated by the two approaches. Synchrotron allows to identify static water molecule, thus the maximum of the density of Figure SI7 concerns water molecules strongly bound to Cu. On the contrary, molecular dynamics allows to discover the contribution to the density arising also from more mobile water molecules, e.g., those possibly forming water trimers with H_2O bound to Cu. Of course, the mismatch may partly arise also from a limited accuracy of the force field used in the simulations, which has not been optimized for this work.

Summarizing, molecular dynamics, liquid porosimetry and synchrotron results suggest that while the MOF is overall hydrophobic, locally there are attractive interactions that determine its properties, such as the observed strong reduction of the critical temperature.

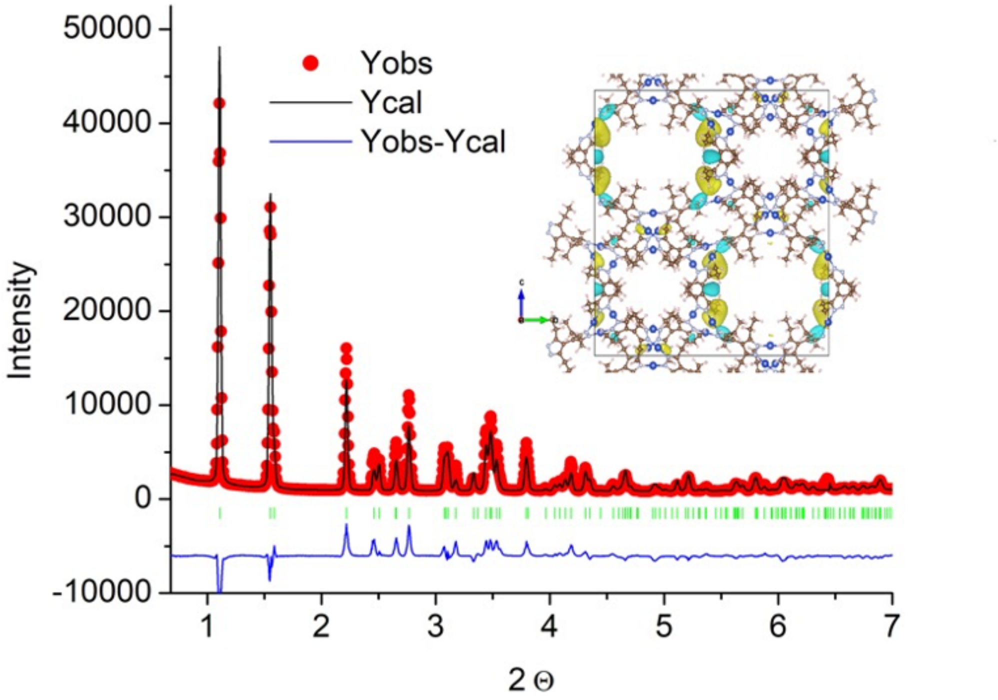

Figure SI7. Full profile refinement of the wet Cu_2(tebpz) at 0.4 MPa and 278.15 K at the end of the first decompression. Inset presents respective residual difference density at 0.11 isosurface level. One can identify remnant water positions close to triangular groups of Cu atoms.

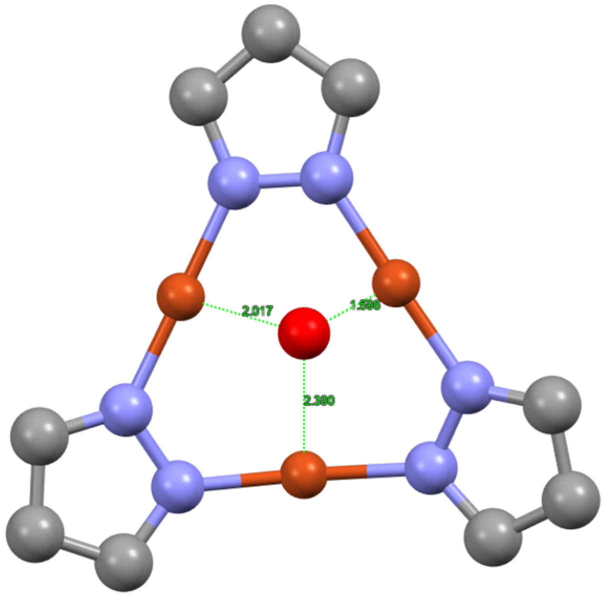

Figure SI8. Most probable water site locates in the vincinity of the lateral apertures close to the triple Cu(I) sites. The distances listed in the Figure are approximately 1.6, 2.0 and 2.4 Å.

Figure SI9 presents Le Bail fit of Cu_2(tebpz) at 35 MPa and 428.15 K in the extruded state, after pressurization at 35 MPa. Same as at lower pressures, one sees no additional peaks,

confirming the preservation of the crystalline structure and no emergence of other phases. As far as lattice parameters are concerned, the fitted values are (in Å): a = 10.3576(2), b = 32.5751(6), 33.3482(5). Once again, changes in the lattice parameters are limited, with some of the expanding and other contracting. In all cases, these changes are insufficient to justify the significant drop of the intruded volume, that is almost negligible at this temperature. This fact, accompanied by the reduction of the hydrophobicity with increasing temperature, determined both experimentally and theoretically, provides further evidence for the occurrence of a supercritical transition at very mild thermodynamic conditions. Figure SI10 shows the system after pressure has been reduce to 1 MPa, keeping the temperature constant at 428.15 K. Like at all thermodynamic conditions considered, there is no evidence of the emergence of new phases or any structural change of Cu_2(tebpz). Here, lattice parameters are (in Å): a = 10.3830(2), b = 32.5460(6), 33.4693(5), once again in line with values reported at the other thermodynamic conditions.

In conclusion, synchrotron data show that over the entire range of temperatures and pressure considered i) the *Pnnn* symmetry of the MOF structure is preserved, ii) no extra phases is present in the sample, and iii) changes in the lattice parameter is largely insufficient to explain the change in the intrusion volume. Moreover, iv) Fourier difference maps show an excess of electronic density that can be explained by a high dense vapor phase within the elliptical cavities of Cu_2(tebpz), which is consistent with molecular dynamics predictions. Remarkably, v) the regions of high density as identified in synchrotron experiments are coherent with computational results. In particular, vi) water is found at the apertures on the lateral walls of MOF's cavities, in correspondence of copper atoms.

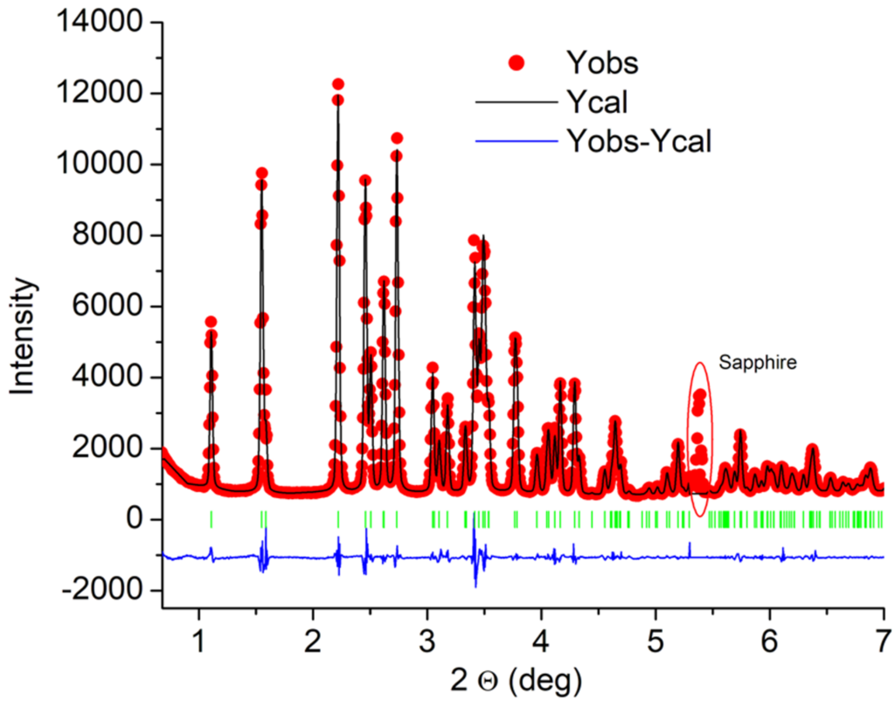

Figure SI9. Le Bail fit Cu_2(tebpz) at 35 MPa and 428.15 K.

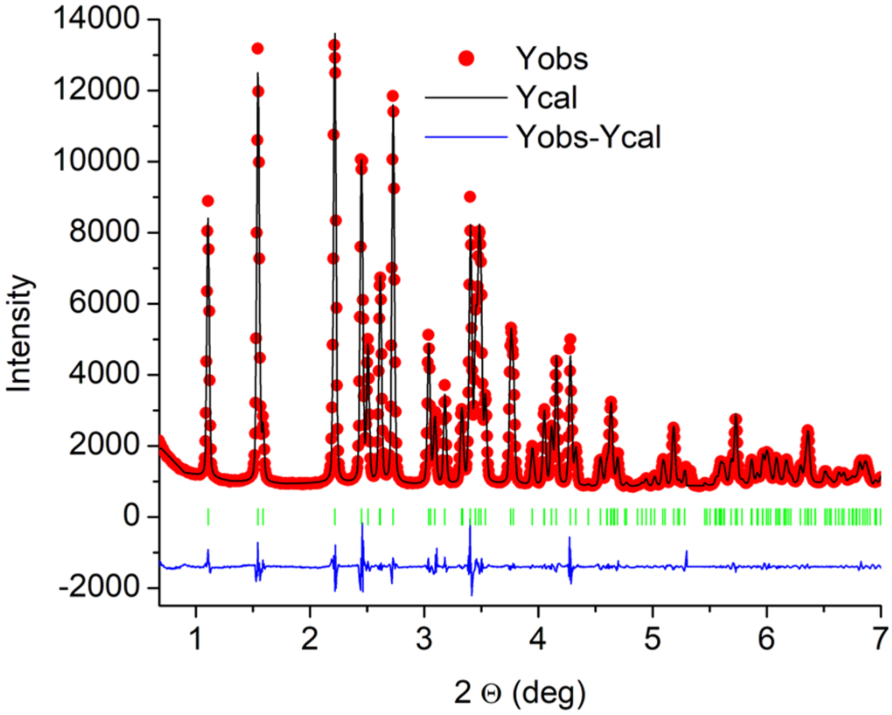

Figure SI10. Le Bail fit Cu_2(tebpz) at 1 MPa and 428.15 K, after pressurization at 35 MPa.

Section SI2 - {Cu_2(tebpz)+water} model.

The computational sample consists of a 4-unit-cell-thick slab of Cu_2(tebpz) (~4.2 nm) and ~2000 water molecules, for a total (Cu_2(tebpz)+ H_2O) of ~10700 atoms. Periodic boundary conditions were applied along the *b*, *c* lattice directions while a pair of pistons were introduced to control the pressure applied to the liquid along the *a* direction, which is the same direction in which the MOF unit-cell is replicated 4-fold (Figure SI11). The partial charges of the atoms were calculated by ab initio methods (Bader charges/Lowdin charges comparison) with QUANTUM ESPRESSO,[4] while the force field for the MOF were generated using UFF4MOF.[5]

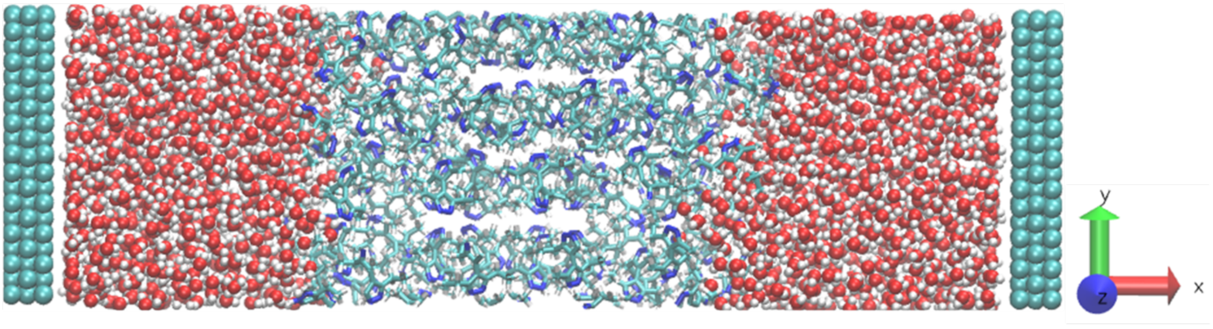

Figure SI11. Computational model of Cu_2(tebpz) + water slab. One can notices the two piston that exert pressure direct on the thick films of water in both directions.

Considering that our MOF is highly hydrophobic, in order to study the intruded system, we decided to proceed with a brute-force technique: a high pressure of 200MPa was applied via pistons to regions of liquid water, which promoted and achieved the intrusion of the liquid into

the cavities of the MOF (Figure SI12). This technique allowed us to obtain the intruded system in an equilibrium state in a time consonant with typical classical MD simulations, tens of ns. Once intruded, we brought the system to conditions as closed as possible to those of the experimental analysis: the pressure is then relaxed down to 25 MPa, slightly above the experimental value of intrusion pressure at 300K. The temperature is then slowly increased from 300 to 400K. In this *in silico* experiment we observe a spontaneous extrusion on the nanosecond time scale at 360-370 K (Figure SI13) which is consistent with present and previous experiment.[6] This can be attributed to an increase of the intrusion pressure with temperature. Thus, though we refrain from claiming that the computational setup guarantees a quantitative prediction of the intrusion/extrusion characteristics of the system, our simulations capture the key aspects of the {Cu_2(tebpz) +water} system.

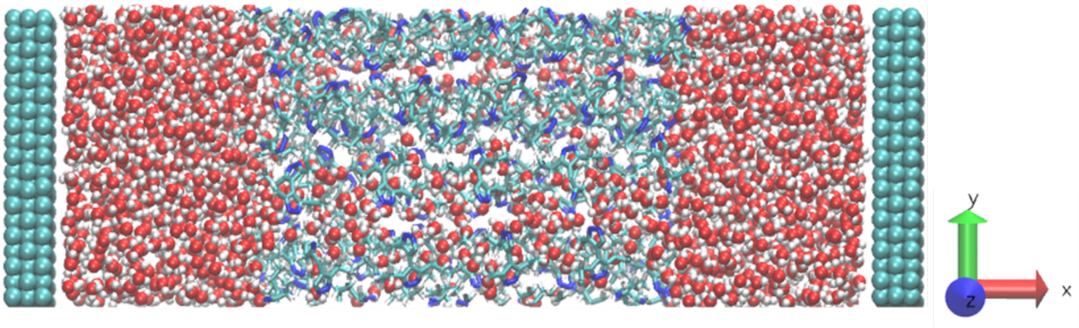

Figure SI12. Cu_2(tebpz) + water slab during water intrusion. The intrusion was achieved by two pistons exerting a pressure of 200 MPa on the water.

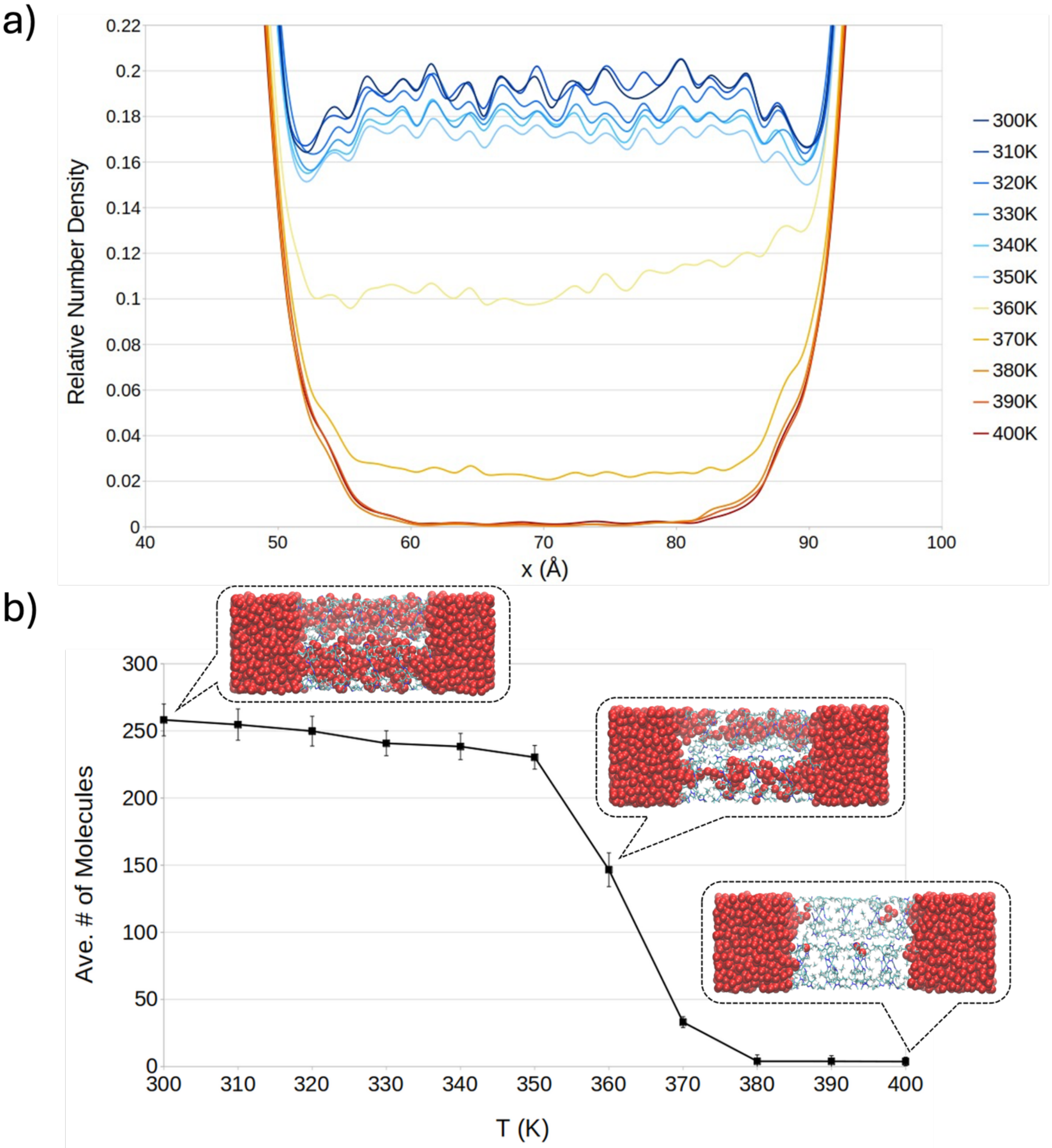

Figure SI13. a) Relative number density profiles at 25MPa as a function of temperature, normalized with respect to bulk water. One observes a sharp change in the density profile at 360K, corresponding to temperature-induced extrusion, which is consistent with present results on the intrusion (and extrusion) pressure(s) as well as previous results for temperature-driven extrusion of Cu_2(tebpz).[6] This is highlighted by the average number of liquid-like water molecules intruded into the MOF as a function of temperature (panel b). Screenshots of the oxygen atoms (red) of the water molecules occupying the elliptical channels of the MOF at temperatures of 300, 360, 400K are also shown. The temperature is increased in successive steps of 10 K and kept constant for 5 ns.

Section SI3 - Cu_2(tebpz) circular channels water density profile.

From the density profiles, in the two 6.2 Å diameter cylindrical channels, there is no consistent presence of vapor-like water molecules, but rather highly mobile vapor molecules as observed from the MD trajectories. By increasing the temperature (440 K) and the applied pressure (from 25 to 45 MPa), there is a slight increase in the number of molecules (Figure SI14). However, this does not have a major influence on the estimation of the density field of the confined water molecules.

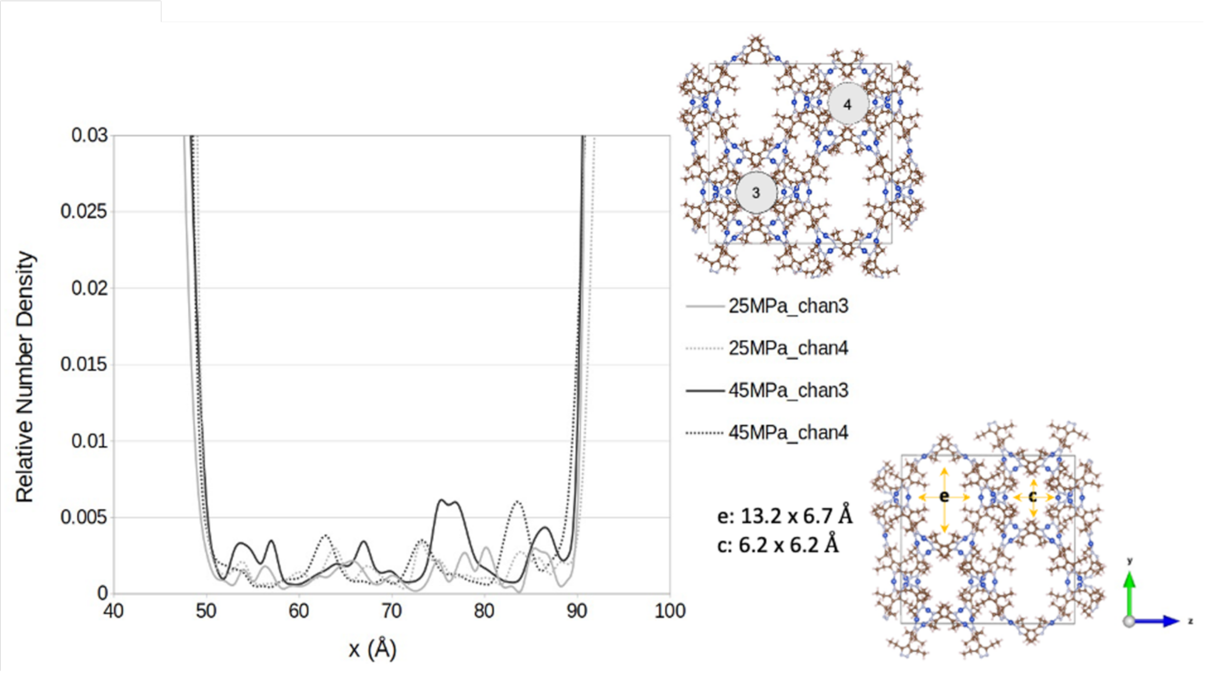

Figure SI14. Elliptical channels vapor density profiles comparison 25 vs 45 MPa at 440 K, normalized with respect to bulk water (number density equal to 1).

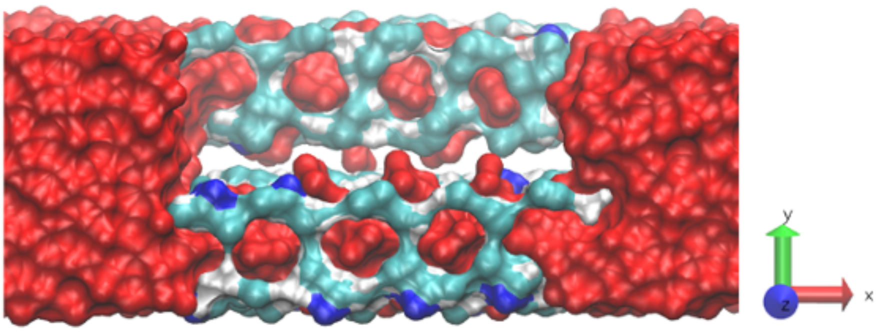

Figure SI15. Snapshot of intruded MOF, both elliptical channels (colored) and water (red) are depicted as "surfaces" in order to highlight the peculiar morphology of the material and intruded state, such as secondary porosity and the structural origins of inhomogeneous nature of density profiles.

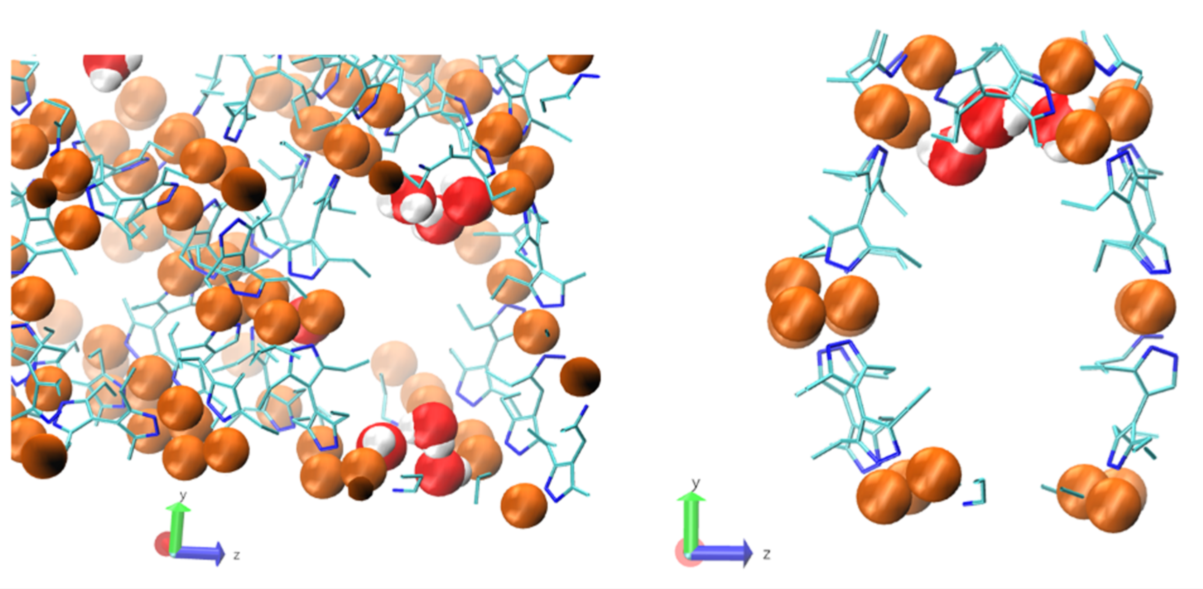

Figure SI16. Snapshots of vapor-like water trimers present inside the elliptical channels and located near the Cu atoms of secondary porosity of the MOF, obtained from MD trajectory at 50MPa and 480K (near Tc).

Section SI4 - Effect of temperature on {ZIF-8 + water}.

In previous works[7,8] Grosu et al. have reported a trend of intruded volume with *T* analogous to that observed for Cu_2(tebpz) (Figure SI17). These authors also noticed a sizable reduction of the intrusion volume within the temperature range investigated. The latter is smaller than the temperature range considered for Cu_2(tebpz) due to the limited thermal stability of ZIF-8, which we believe prevented to observe the disappearance of intrusion altogether. However, akin to Cu2(tebpz), in Figure SI18 we reported the trend of the first derivative of the intrusion pressure with respect to the intrusion volume as a function of temperature (obtained from PV isotherm curves). The decreasing linear trend is significant of a decrease in the intrusion volume of ZIF-8 with temperature.

To test whether the significant reduction of the intruded volume with temperature in ZIF-8-based HLS can be ascribed to an incipient supercritical transition, we performed simulations analogous to those of {Cu_2(tebpz) + water}. Starting with an intruded ZIF-8 at 300 K and 25 MPa, extensively investigated in previous articles,[9,10] we increased the temperature in steps of 20 K, observing a continuous reduction of the density, which becomes even more marked from 400 K onwards (Figure SI19). At 460 K the density ceases to significantly decrease, remaining almost constant up to 600 K, when we stopped our *in silico* experiment. Here, we started decreasing the temperature down to 300 K. As for Cu_2(tebpz), and consistent with experiments, at lower temperatures the system supports two states: intruded, filled with liquid, and extruded, filled with vapor (in the case of our simulations where we have no insoluble gasses). Like for Cu_2(tebpz), the density of gas sizably increases with temperature, the density of the liquid-like and gas-like phases becomes almost equal at 500 K. However, as opposed to Cu_2(tebpz), the difference between two densities is beyond the statistical error. We postulate that this is due to metastabilities of ZIF-8: for water density to increase/decrease H_2O molecules have to pass through 0.35 nm-narrow apertures connecting the MOF's cavities and our simulations may be too short to reach equilibrium within the long but possibly insufficient simulation time. Indeed, while Cu_2(tebpz) shows negligible intrusion/extrusion hysteresis, the same phenomenon is

significant in ZIF-8[11] even on the seconds/minutes experimental timescale, a timescale ~8 orders of magnitude longer that the one achievable in atomistic simulations.

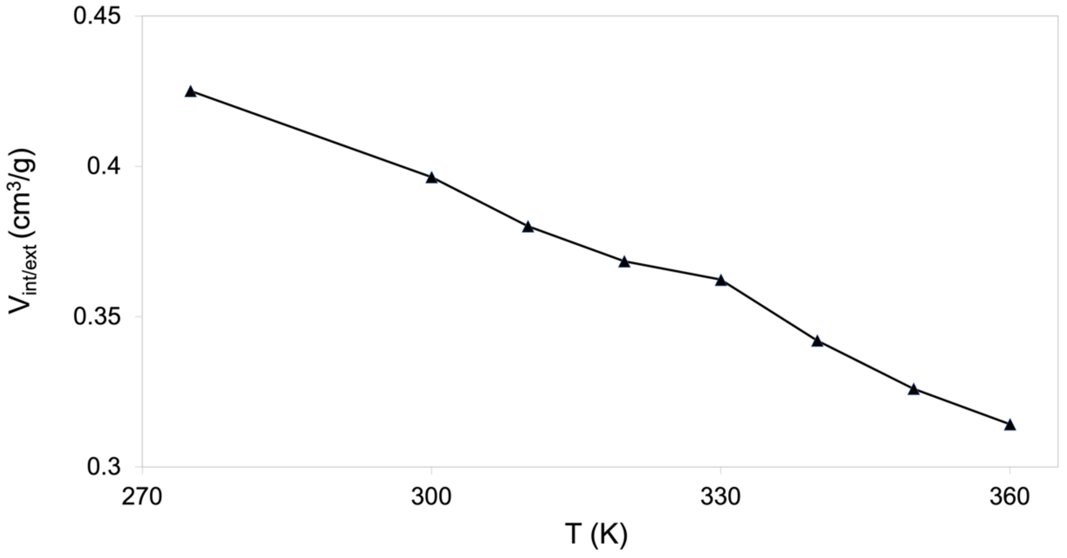

Figure SI17. Dependence of the intrusion volume as a function of the temperature for ZIF-8.2 Used with permission of RSC, from Synergetic Effect of Temperature and Pressure on Energetic and Structural Characteristics of {ZIF-8 + Water} Molecular Spring, Grosu, Y., Renaudin, G., Eroshenko, V., Nedelec, J. M., Grolier, J. P. E., Nanoscale 2015, 7 (19), 8803–8810; permission conveyed through Copyright Clearance Center, Inc.

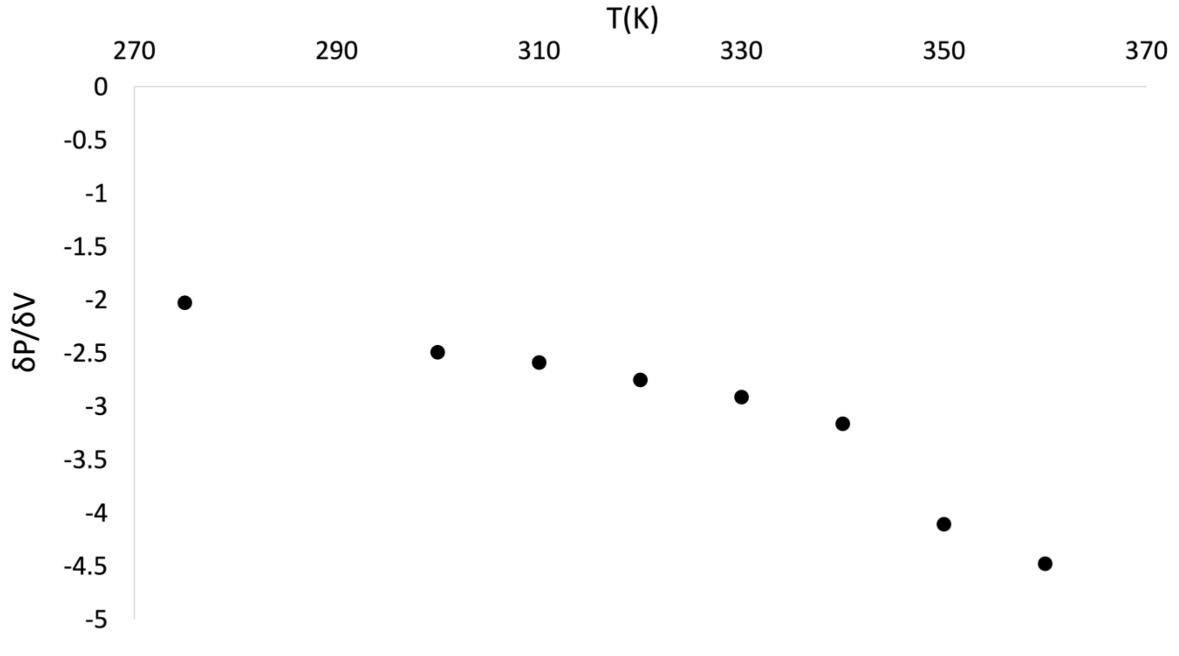

Figure SI18. Trend of the slope of PV isotherms for ZIF-8 obtained in 275K-360K temperature range (or in the operative experimental temperature range). A change of slope is perceived at ~340 K but the limited range of stability of ZIF-8 prevented us to explore the trend of the slope on a larger interval and arrive at reliable conclusions about T_c possibly even lower than for Cu_2(tebpz).

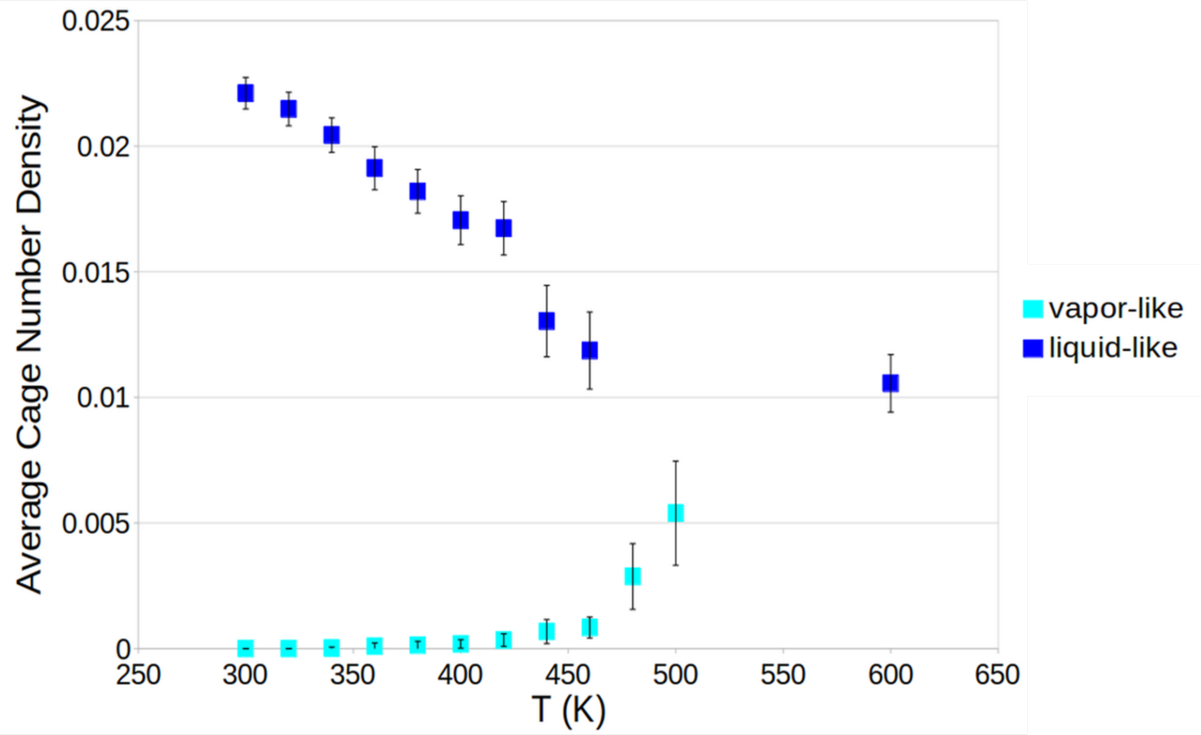

Figure SI19. Normalized average number density of liquid/vapor-like water confined in ZIF-8.

REFERENCES

(1) Amayuelas, E.; Tortora, M.; Bartolomé, L.; Littlefair, J. D.; Paulo, G.; Le Donne, A.; Trump, B.; Yakovenko, A. A.; Chorążewski, M.; Giacomello, A.; Zajdel, P.; Meloni, S.; Grosu, Y. Mechanism of Water Intrusion into Flexible ZIF-8: Liquid Is Not Vapor. *Nano Lett* **2023**, *23* (12), 5430–5436. https://doi.org/10.1021/acs.nanolett.3c00235.

(2) Wang, J. H.; Li, M.; Li, D. An Exceptionally Stable and Water-Resistant Metal-Organic Framework with Hydrophobic Nanospaces for Extracting Aromatic Pollutants from Water. *Chemistry - A European Journal* **2014**, *20* (38), 12004–12008. https://doi.org/10.1002/chem.201403501.

(3) S-Y Chui, S.; M-F Lo, S.; H Charmant, J. P.; Guy Orpen, A.; Williams, I. D. A Chemically Functionalizable Nanoporous Material [Cu3(TMA)2(H2O)3] n. *Science (1979)* **1999**, *283* (5405), 1148–1150. https://doi.org/10.1126/science.283.5405.1148.

(4) Giannozzi, P.; Andreussi, O.; Brumme, T.; Bunau, O.; Buongiorno Nardelli, M.; Calandra, M.; Car, R.; Cavazzoni, C.; Ceresoli, D.; Cococcioni, M.; Colonna, N.; Carnimeo, I.; Dal Corso, A.; de Gironcoli, S.; Delugas, P.; Distasio, R. A.; Ferretti, A.; Floris, A.; Fratesi, G.; Fugallo, G.; Gebauer, R.; Gerstmann, U.; Giustino, F.; Gorni, T.; Jia, J.; Kawamura, M.; Ko, H. Y.; Kokalj, A.; Kücükbenli, E.; Lazzeri, M.; Marsili, M.; Marzari, N.; Mauri, F.; Nguyen, N. L.; Nguyen, H. v.; Otero-De-La-Roza, A.; Paulatto, L.; Poncé, S.; Rocca, D.; Sabatini, R.; Santra, B.; Schlipf, M.; Seitsonen, A. P.; Smogunov, A.; Timrov, I.; Thonhauser, T.; Umari, P.; Vast, N.; Wu, X.; Baroni, S. Advanced Capabilities for Materials Modelling with Quantum ESPRESSO. *Journal of Physics Condensed Matter* **2017**, *29* (46). https://doi.org/10.1088/1361-648X/aa8f79.

(5) Coupry, D. E.; Addicoat, M. A.; Heine, T. Extension of the Universal Force Field for Metal-Organic Frameworks. *J Chem Theory Comput* **2016**, *12* (10), 5215–5225. https://doi.org/10.1021/acs.jctc.6b00664.

(6) Chorążewski, M.; Zajdel, P.; Feng, T.; Luo, D.; Lowe, A. R.; Brown, C. M.; Leão, J. B.; Li, M.; Bleuel, M.; Jensen, G.; Li, D.; Faik, A.; Grosu, Y. Compact Thermal Actuation by Water and Flexible Hydrophobic Nanopore. *ACS Nano* **2021**, *15* (5), 9048–9056. https://doi.org/10.1021/acsnano.1c02175.

(7) Grosu, Y.; Renaudin, G.; Eroshenko, V.; Nedelec, J. M.; Grolier, J. P. E. Synergetic Effect of Temperature and Pressure on Energetic and Structural Characteristics of {ZIF-8 + Water} Molecular Spring. *Nanoscale* **2015**, *7* (19), 8803–8810. https://doi.org/10.1039/c5nr01340b.

(8) Grosu, Y.; Eroshenko, V.; Nedelec, J. M.; Grolier, J. P. E. A New Working Mode for Molecular Springs: Water Intrusion Induced by Cooling and Associated Isobaric Heat Capacity Change of a {ZIF-

8 + Water} System. *Physical Chemistry Chemical Physics* **2015**, *17* (3), 1572–1574. https://doi.org/10.1039/c4cp03944k.

(9) Tortora, M.; Zajdel, P.; Lowe, A. R.; Chorążewski, M.; Leão, J. B.; Jensen, G. V.; Bleuel, M.; Giacomello, A.; Casciola, C. M.; Meloni, S.; Grosu, Y. Giant Negative Compressibility by Liquid Intrusion into Superhydrophobic Flexible Nanoporous Frameworks. *Nano Lett* **2021**, *21* (7), 2848–2853. https://doi.org/10.1021/acs.nanolett.0c04941.

(10) Zajdel, P.; Madden, D. G.; Babu, R.; Tortora, M.; Mirani, D.; Tsyrin, N. N.; Bartolomé, L.; Amayuelas, E.; Fairen-Jimenez, D.; Lowe, A. R.; Chorążewski, M.; Leao, J. B.; Brown, C. M.; Bleuel, M.; Stoudenets, V.; Casciola, C. M.; Echeverría, M.; Bonilla, F.; Grancini, G.; Meloni, S.; Grosu, Y. Turning Molecular Springs into Nano-Shock Absorbers: The Effect of Macroscopic Morphology and Crystal Size on the Dynamic Hysteresis of Water Intrusion-Extrusion into-from Hydrophobic Nanopores. *ACS Appl Mater Interfaces* **2022**, *14* (23), 26699–26713. https://doi.org/10.1021/acsami.2c04314.

(11) Sun, Y.; Rogge, S. M. J.; Lamaire, A.; Vandenbrande, S.; Wieme, J.; Siviour, C. R.; Van Speybroeck, V.; Tan, J. C. High-Rate Nanofluidic Energy Absorption in Porous Zeolitic Frameworks. *Nat Mater* **2021**, *20* (7), 1015–1023. https://doi.org/10.1038/s41563-021-00977-6.
